# The Impact of Digital Health Interventions on Psychological Health, Self-Efficacy, and Quality of Life in Patients With End-Stage Kidney Disease: Systematic Review and Meta-Analysis

**DOI:** 10.2196/74414

**Published:** 2025-09-26

**Authors:** Jiayi Zhu, Jianfei Xie, Yating Luo, Xiaoqian Dong, Zitong Lu, Huiyi Zhang, Jingying Wang, Min Liu, Andy SK Cheng

**Affiliations:** 1 Nursing Department, The Third Xiangya Hospital, Central South University, 138 Tongzipo Road, Yuelu District, Changsha, Hunan, 410013, China; 2 Xiangya School of Nursing, Central South University, Changsha, China; 3 Research Center for Clinical Nursing Safety Management, Central South University, Changsha, China; 4 School of Health Sciences, Western Sydney University, Sydney, Australia

**Keywords:** digital health interventions, psychological health, self-efficacy, quality of life, end-stage kidney disease, systematic review

## Abstract

**Background:**

End-stage kidney disease (ESKD) imposes a significant global health burden, with patients often experiencing poor quality of life (QoL) due to psychological distress and low self-efficacy. Digital health interventions (DHIs) offer potential to address these challenges. However, their effects in this population remain inconsistent, and a comprehensive synthesis of the evidence is lacking.

**Objective:**

The present study aims to assess the impact of DHIs on the psychological health, self-efficacy, and QoL of patients with ESKD and to evaluate engagement, adherence, and satisfaction with these interventions.

**Methods:**

A comprehensive search was conducted across six electronic databases (PubMed, Web of Science, Cochrane Library, PsycINFO, Embase, and CINAHL) up to January 21, 2025. Randomized controlled trials (RCTs) examining the effects of DHIs on psychological health, self-efficacy, or QoL in patients with ESKD were included. Two reviewers independently screened studies, extracted data, and assessed the risk of bias using the Cochrane Risk of Bias Tool (RoB 2). A meta-analysis was performed using Review Manager 5.4, with subgroup analyses by treatment modality, intervention type, and duration. Evidence quality was assessed using the Grading of Recommendation, Assessment, Development, and Evaluation (GRADE) approach.

**Results:**

Twenty-three RCTs involving 2407 patients with ESKD from 12 countries were included. DHIs significantly improved depression (standardized mean differences [SMD] −0.41, 95% CI −0.63 to −0.19, *P*=.003) and overall QoL (SMD 0.55, 95% CI 0.07-1.03, *P*=.03). While DHIs did not significantly improve overall self-efficacy (SMD 0.56, 95% CI −0.06 to 1.18, *P*=.08), a benefit was observed in patients on hemodialysis (SMD 0.59, 95% CI 0.34-0.83, *P*<.001). Engagement was favorable, with completion rates above 63%, adherence rates of 54%‐79%, and generally positive patient feedback on DHIs. Application-based interventions improved self-efficacy (SMD 0.66, 95% CI 0.31-1.02, *P*<.001) and overall QoL (SMD 0.50, 95% CI 0.04-0.96, *P*=.003); telemedicine improved depression (SMD −0.88, 95% CI −1.21 to −0.56, *P*<.001) and self-efficacy (SMD 2.76, 95% CI 2.32-3.20, *P*<.001); and video-based interventions improved depression (SMD -0.34, 95% CI −0.55 to −0.13, *P*=.002) and overall QoL (SMD 0.31, 95% CI 0.15-0.46, *P*<.001). Due to high heterogeneity and risk of bias, evidence quality was rated as low for depression and overall QoL, moderate for general anxiety, and very low for stress and self-efficacy.

**Conclusions:**

DHIs can significantly improve the psychological health and QoL of patients with ESKD, particularly when tailored to patients’ needs and delivered through interactive platforms such as apps and telemedicine. High engagement and positive patient feedback suggest good acceptability in clinical practice. However, low evidence quality warrants cautious interpretation. Future research should involve more high-quality RCTs and design DHIs that address the unique needs of older patients, patients on peritoneal dialysis, and kidney transplant recipients.

## Introduction

### Background

Chronic kidney disease is a global health burden, affecting approximately 850 million people worldwide [1]. As the population ages and the prevalence of chronic diseases like hypertension and diabetes rises, the incidence of end-stage kidney disease (ESKD) continues to increase [2,3]. Projections suggest that from 2015 to 2030, ESKD prevalence in the United States will rise by 29%-68% [4]. Despite improvements in renal replacement therapy, dialysis technology, and nursing care, the quality of life (QoL) for patients with ESKD remains significantly reduced [5-7]. Patients with ESKD experience severe symptoms, economic strain, and psychological challenges, leading to poor QoL. All aspects of QoL in patients with ESKD are notably diminished when compared with the general population [8]. Psychological health and self-efficacy have been identified as key factors influencing QoL in these patients.

Psychological issues such as depression, anxiety, and stress are closely linked to poorer clinical outcomes and diminished QoL in patients with ESKD [1,9,10]. Although renal replacement therapies alleviate some disease symptoms, patients often face depression and anxiety due to economic burdens, dialysis-related side effects, and lack of rehabilitation knowledge [7]. Poor psychological health can reduce treatment adherence, worsen physical symptoms, and lead to adverse outcomes such as disease progression, graft loss, and death, further impacting QoL [11,12]. Additionally, mental health challenges can hinder self-management motivation and reduce patients’ autonomy in managing their disease, making it more difficult to engage in health-promoting behaviors and worsening health-related QoL [13].

Self-efficacy, defined as one’s belief in their ability to perform tasks and achieve goals [14], is positively correlated with QoL [9,12,15]. Studies have shown that patients with ESKD with higher self-efficacy are more confident in self-care and disease management, exhibit better treatment adherence, and are more likely to adopt new strategies to improve their health, leading to better physical health and enhanced QoL [9,13,16]. However, many patients with ESKD have low self-efficacy due to factors such as treatment complexity, lack of knowledge, poor psychological status, and insufficient social support [17,18].

Given the poor QoL of many patients with ESKD, there is an urgent need for effective and accessible interventions. Traditional face-to-face interventions often require significant resources, involve time and spatial constraints, and may increase the burden on patients. Digital health interventions (DHIs) have emerged as a promising solution, offering a more convenient and scalable alternative.

DHIs utilize digital technologies such as apps, telemedicine, videos, virtual reality (VR), and wearable devices to provide health support, education, monitoring, and treatment [19,20]. These interventions can overcome geographical barriers, reach a broader population, and offer personalized recommendations based on individual health data, thus improving self-management and health literacy in patients with chronic disease [21-23]. Previous studies have shown that DHIs can improve QoL, self-management abilities, and treatment adherence in patients with chronic kidney disease [24-26]. However, their effectiveness in patients with ESKD remains uncertain.

The field of DHI has seen significant progress in technology and intervention design. However, early studies, often reliant on basic technologies and driven by commercial interests, frequently overlooked patient technological literacy, cultural context, and interactivity [27,28], potentially failing to reflect current advancements or best practices. The COVID-19 pandemic has accelerated the adoption of DHI solutions, but it has also highlighted the limitations of certain interventions [29], such as disparities in technological knowledge, unresolved digital divides among older patients, and limited access to digital devices in socioeconomically disadvantaged populations [30,31]. Additionally, conflicting findings on the efficacy of DHIs in improving psychological health, self-efficacy, and QoL in patients with ESKD, coupled with methodological challenges (eg, small sample sizes, high risk of bias) [32-34], have contributed to a lack of consensus on their effectiveness. To date, no comprehensive review has quantitatively assessed the impact of DHI on psychological health, self-efficacy, and QoL in patients with ESKD.

### Goal of This Study

This systematic review and meta-analysis aims to (1) summarize the types and content of DHIs designed to improve psychological health, self-efficacy, and QoL in patients with ESKD and analyze patient engagement and experience; (2) quantitatively evaluate their effectiveness and analyze effective intervention formats; and (3) provide recommendations for future research.

## Methods

This systematic review and meta-analysis followed the Cochrane Handbook for Systematic Reviews of Interventions [35] and reported the results according to the PRISMA (Preferred Reporting Items for Systematic Reviews and Meta-Analyses) 2020 guidelines [36]. The protocol was registered on PROSPERO (Registration number: CRD42024629357). A completed PRISMA 2020 checklist is provided as Checklist 1.

### Data Sources and Search Strategies

A comprehensive literature search was conducted up to January 21, 2025, in six databases: PubMed, Web of Science, Cochrane Library, PsycINFO, Embase, and CINAHL. The search strategy was developed by the researchers in collaboration with a librarian and adapted for each database, including combinations of Medical Subject Headings terms and keywords in PubMed. The search terms included (1) end-stage kidney disease, (2) digital health, (3) psychological health, (4) self-efficacy, and (5) quality of life. In addition, we manually searched the reference lists of identified articles and performed citation tracking of relevant systematic reviews. We reran the database search before the final data analysis to include any new studies. Gray literature, such as conference abstracts and dissertations, was not included. The full search strategy is provided as Multimedia Appendix 1.

### Inclusion Criteria

The study population included adults (≥18 y) diagnosed with ESKD, defined as a glomerular filtration rate <15 ml/min/1.73 m² [37], including those receiving kidney replacement therapy (hemodialysis [HD], peritoneal dialysis [PD], and kidney transplantation [KT]) or conservative (nondialysis) management.

The intervention used was DHI delivered via digital platforms or technologies, including but not limited to mobile apps, telemedicine, video, social media, wearable devices, online platforms, and virtual technologies. The intervention must aim to improve psychological health, self-efficacy, or QoL in patients with ESKD.

The comparison group consisted of standard medical care, such as regular dialysis care, routine counseling, or standard health education programs, without digital components, or an alternative group as defined in the original study.

Studies should report one or more of the following outcomes: psychological health (including depression, anxiety, stress), self-efficacy, and QoL.

The study type used was randomized controlled trials (RCTs).

### Exclusion Criteria

The exclusion criteria included (1) studies published before January 1, 2019, to ensure relevance and timeliness of the evidence; (2) conference abstracts, conference proceedings, or other gray literature; (3) protocols or trial registration records only; and (4) non-English publications.

### Study Selection and Data Extraction

All retrieved studies were imported into Endnote 21 reference management software and duplicates were removed. Two reviewers (JZ and ML) independently screened titles and abstracts for eligibility based on the inclusion and exclusion criteria. Full texts of potentially relevant studies were reviewed to select studies meeting the criteria, and reasons for exclusion were documented. Any disagreements were resolved through discussion or by consulting a third reviewer (XD).

Data extraction was independently performed by the same two reviewers (JZ and ML) using a structured form. The form included details such as the first author, publication year, country, patient type, sample size, control methods, a brief description of the intervention, outcome measures, and intervention completion rate. The completion rate of interventions across all studies was evaluated by calculating the ratio of participants who completed the post-intervention assessment to the total number of participants initially enrolled in the intervention. If necessary data were missing, corresponding authors were contacted by email. A third reviewer (XD) verified the extracted data.

To comprehensively capture the diversity of DHIs, we classified each intervention based on its core approach and content. This classification was inductively developed by two reviewers (JZ and ML) through full-text review of the included studies. Any disagreements in classification were resolved through discussion or consultation with a third reviewer (XD).

### Risk of Bias Assessment

Two reviewers (JZ and ML) independently assessed the methodological quality of included RCTs using the Cochrane Risk of Bias Tool (RoB 2) [38]. The tool evaluates risk in five domains: randomization process, deviations from intended interventions, missing outcome data, measurement of the outcome, and selection of reported results. Each study was rated as low risk, some concerns, or high risk. Disagreements were resolved through discussion or consultation with a third reviewer, and kappa coefficients were calculated to assess inter-rater consistency (XD).

### Quality of Evidence Evaluation

The Grading of Recommendation, Assessment, Development, and Evaluation (GRADE) system was used to assess the quality of the evidence. Evidence from RCTs was initially rated as high quality, with potential downgrades based on five factors: risk of bias, inconsistency, indirectness, imprecision, and other considerations (including publication bias, large effect, plausible confounding, and dose-response gradient). Publication bias was assessed using visual inspection of funnel plots when ≥10 studies were available for an outcome. When fewer than 10 studies were available, publication bias was not formally assessed due to the low statistical power of asymmetry tests; in such cases, no downgrade was applied for publication bias in the GRADE assessment unless other evidence suggested its presence. Evidence quality was classified as high, moderate, low, or very low. Two reviewers (JZ and ML) independently conducted the GRADE assessment using the GRADEpro GDT tool, followed by a third reviewer (XD) to resolve discrepancies.

### Data Synthesis and Analysis

Meta-analysis and heterogeneity tests were performed using Review Manager 5.4 software. Due to the use of different outcome measures across studies, standardized mean differences (SMD) with 95% CIs were used to report effect sizes. A random-effects model was employed due to expected heterogeneity across studies in terms of interventions, populations, and outcome measures. *I*² statistics and *P* values were calculated to assess heterogeneity. Subgroup analyses were conducted based on treatment modality, intervention type, and duration, as these variables directly reflect the clinical heterogeneity of patients with ESKD, differences in intervention technical characteristics, and the ongoing debate on dose-response effects. Sensitivity analysis using the one-study-out method was performed to test the robustness of the combined results. Statistical significance was set at *P*<.05.

## Results

### Search Results and Study Selection

**Figure 1.**
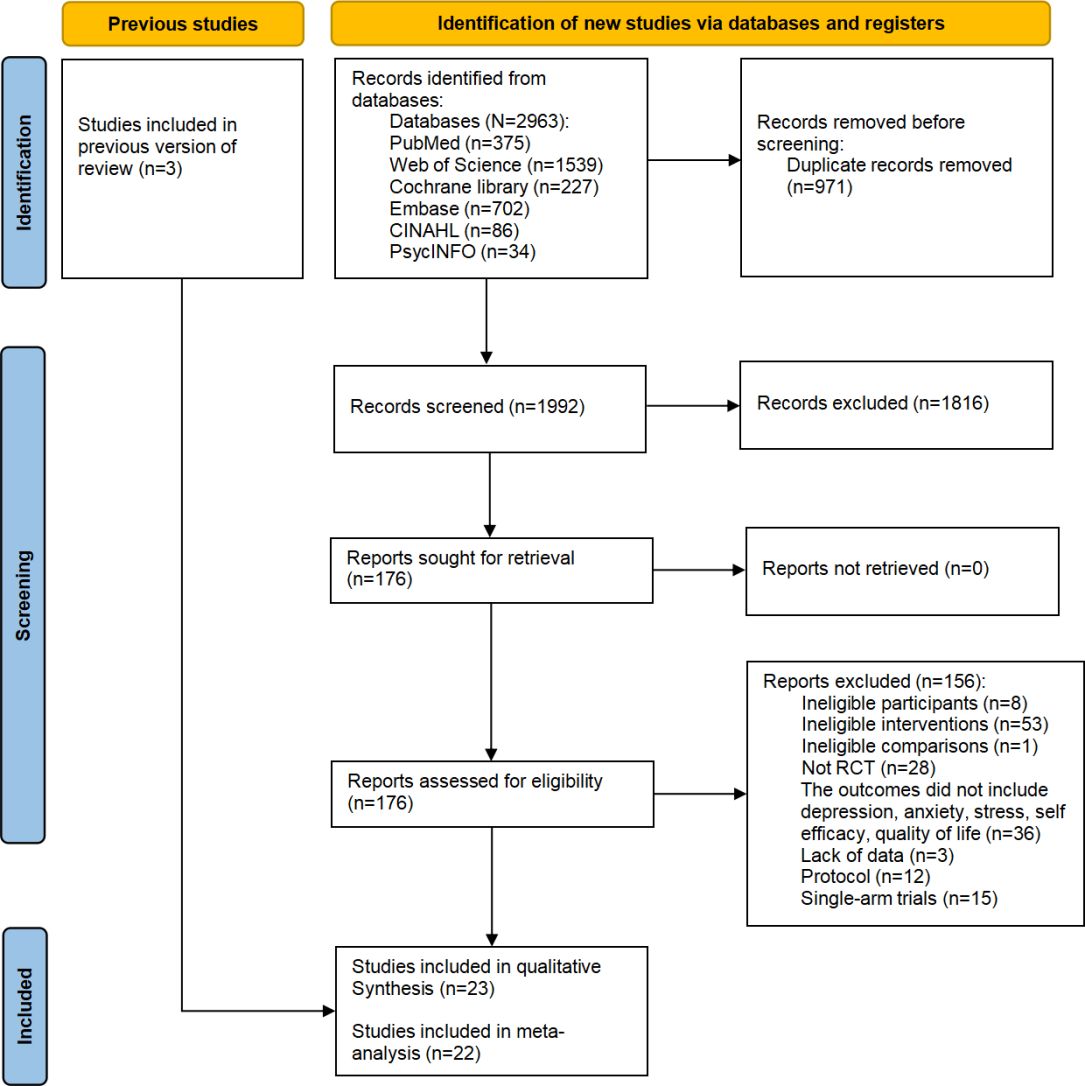
PRISMA flow diagram.

A total of 2963 studies were identified from six electronic databases, of which 971 were duplicates. After screening titles and abstracts, 1816 studies were excluded. Full texts of the remaining 176 studies were assessed for eligibility, and 20 studies met the inclusion criteria. The most common reason for exclusion at the full-text stage was ineligible interventions that did not involve digital platforms or technologies (n=53; eg, traditional face-to-face counseling) [39]. Additionally, three relevant studies were identified from previously published systematic reviews. In total, 23 studies were included in this systematic review [32-34,40-59]. The study selection process is shown in the PRISMA flow diagram (Figure 1).

### Study Characteristics

The 23 included studies were RCTs published between 2019 and 2024 across 12 countries. The distribution of studies by country was as follows: China (4/23, 17.4%) [40,47,49,59], Korea (3/23, 13%) [34,44,50], Iran (3/23, 13%) [45,55,57], the United States (2/23, 8.7%) [43,54], Canada (2/23, 8.7%) [42,51], Australia (2/23, 8.7%) [52,53], Brazil (2/23, 8.7%) [56,58], Turkey (1/23, 4.3%) [33], Thailand (1/23, 4.3%) [41], Singapore (1/23, 4.3%) [32], the Netherlands (1/23, 4.3%) [48], and Poland (1/23, 4.3%) [46]. Detailed characteristics of the included studies are presented in Table 1.

**Table 1.** Characteristics of included studies.

Source	Country	Type of patients	Sample size		Control	Intervention		Outcomes (measures)	Intervention completion rate(%)
			Experimental	Control		Brief introduction	Format		
Zhang et al [40], 2024	China	PD^u^	80	80	Usual care	A 6-mo hospital-community online management model involving three components: medication management, specific operations, and follow-up.	Telemedicine	Depression (BDI)^b^; Self-efficacy (GSES)^c^; Quality of life (KDQOL-SF)^d^	100.0
Taşkin Duman et al [33], 2024	Turkey	HD^e^	26	22	Usual care	A 12-wk video-based training program covering self-management education on hemodialysis basics, fluid intake, nutrition, weight control, and exercise.	Videos	Quality of life (SF-36)^f^	96.3
Pungchompoo et al [41], 2024	Thailand	HD	24	30	Usual care	A 6-mo home telemedicine model addressing renal disease and treatment, food and water management, exercise and stress management, and medication management at home.	Telemedicine	Quality of life (the 9-item Thai Health Status Assessment questionnaire)	80.0
Mansell et al [42], 2024	Canada	KT^g^	64	56	Usual care	A 1-y intervention combining standard education with home-based video education and an adherence contract.	Videos	Self-efficacy (GSES)^c^; Quality of life (SF-12)^h^	70.3
Dember et al [43], 2024	USA	HD	319	324	Usual care	Weekly coach-led sessions via video or telephone conferencing lasting 45 to 50 min for 12 wk, focusing on pain coping skills training.	Videos or telephone	Depression (PHQ-9)^i^; Anxiety (GAD-7)^j^; Quality of life (single-item QOL)^k^	81.4
Chae et al [44], 2024	Korea	PD	27	26	Usual care	A 10-wk self-management mobile apps intervention based on social cognitive theory.	app	Self-efficacy (PD-related self-efficacy); Quality of life (KDQOL-36)^l^	96.4
Aw et al [32], 2024	Singapore	PD	11	10	Usual care	A 4-d video-assisted mindfulness training program.	Videos	Anxiety (State and Trait Anxiety Inventory); Stress (PSS)^m^; Self-efficacy (The Self-Efficacy for Managing Chronic Disease);	84.6
Alishahi et al [45], 2024	Iran	HD	36	36	Usual care	A 30-d therapeutic recreation app intervention involving music listening, comedy movie viewing, exercise routines, and educational question-and-answer games.	app	Depression (BDI-II)^n^	92.3
Turoń-Skrzypińska et al [46], 2023	Poland	HD	39	46	Usual care	Use of the NefroVR prototype system for 20-min VR exercises during hemodialysis, 3 times per week for 3 mo.	VR	Depression (BDI); Anxiety (GAD-7)	76.5
Ren et al [47], 2022	China	HD	38	32	Usual care	A 3-mo microvideo health education program.	Videos	Self-efficacy (CDSES)^o^	95.0
Nadort et al [48], 2022	Netherlands	HD	54	68	Usual care	A 10-wk self-guided online problem-solving therapy based on individual evidence.	app	Depression (BDI-II); Anxiety (BAI)^p^; Quality of life (SF-12)^h^	76.1
Lee et al [49], 2022	China	PD	12	11	Usual care	Apart from usual IPD, a minimum of eight additional VR training sessions on PD exchange, with one to two sessions per week provided by an occupational therapist.	VR	Self-efficacy (Chinese-GSE)	63.6
Pack et al [50], 2021	Korea	HD	37	38	Usual care	An 8-wk smartphone app-based dietary self-management program.	app	Self-efficacy (15‐item dietary self‐efficacy questionnaire); Quality of life (KDQOL‐SF)	92.9
Mansell et al [51], 2021	Canada	KT	64	68	Usual care	Electronic video education consisting of six videos, each lasting between 3 and 24 min.	Videos	Self-efficacy (GSES)^c^;Quality of life (SF-12)	78.0
Dingwall et al [52], 2021	Australia	HD	30	58	Usual care	A 3-mo AIMhi Stay Strong app intervention, developed based on the Aboriginal and Islander Mental Health Initiative and motivational care planning.	app	Depression (PHQ-9)	95.1
Dawson et al [53], 2021	Australia	HD	87	43	Usual care	Standard care plus three weekly text messages for 6 mo, providing advice, information, motivation, and support to improve renal dietary behaviors (related to potassium, phosphorus, sodium, and fluid) as well as general healthy eating and lifestyle behaviors.	Text messages	Quality of life (EuroQol-5D)^q^	89.7
Zhou et al [54], 2020	USA	HD	37	36	Usual care	Virtually supervised intradialytic exergame program conducted during routine hemodialysis for 4 wk, three sessions per week, under non-weight-bearing conditions.	VR	Depression (CES-D)^r^	100.0
Naseri-Salahshour et al [55], 2020	Iran	HD	48	46	Waitlist	A 4-wk healthy nutrition training program conducted via a group channel on the Telegram messenger, enabling free exchange of text, audio, and video.	Multimedia	Quality of life (KDQOL-SF)	94.1
Morais et al [56], 2020	Brazil	Dialysis	35	26	Usual care	A 6-wk complementary leisure activity intervention involving comedy movie sessions twice weekly, with each session lasting approximately 2 h.	Videos	Depression (BDI); Anxiety (Hamilton Anxiety Scale); Quality of life (KDQOL-SF)	94.6
Mansouri et al [57], 2020	Iran	KT	39	39	Usual care	A 2-mo interactive multimedia education intervention.	Multimedia	Quality of life (KTQ-25)^s^	97.5
Cho et al [34], 2020	Korea	HD	23	23	Usual care	A 6-wk self-performance management video program.	Videos	Anxiety (S-STAI)^t^	92.0
Ren et al [59], 2019	China	HD	60	59	Usual care	A 3-mo transtheoretical model-based WeChat health education program on self-management.	Social media	Self-efficacy (CDSES)	81.7
Maynard et al [58], 2019	Brazil	HD	20	20	Usual care	A 12-wk progressive endurance and strength exercise program combined with VR games.	VR	Depression (CES-D); Quality of life (KDQOL-SF)	90.9

a PD: peritoneal dialysis.

b BDI: Beck Depression Inventory.

c GSES: Generalized Self-Efficacy Scale.

d KDQOL-SF: Kidney Disease Quality of Life Short Form.

e HD: hemodialysis.

f SF-36: 36-Item Short Form Health Survey.

g KT: kidney transplantation.

h SF-12: 12-Item Short Form Health Survey.

i PHQ-9: Patient Health Questionnaire-9.

j GAD-7: Generalized Anxiety Disorder-7.

k QOL: quality of life.

l KDQOL-36: Kidney Disease Quality of Life 36-Item Short Form

m PSS: Perceived Stress Scale.

n BDI-II: Beck Depression Inventory-II.

o CDSES: Chronic Disease Self-Efficacy Scale.

p BAI: Beck Anxiety Inventory.

q EuroQOL-5D: EuroQol-5 Dimensions.

r CES-D: Center for Epidemiologic Studies Depression Scale.

s KTQ-25: Kidney Transplant Quality of Life 25-Item Questionnaire.

t S-STAI: State-Trait Anxiety Inventory.

### Participant Characteristics

A total of 2407 patients with ESKD were included in this study. Of these, 1210 patients received DHI, while 1197 patients received usual care. The participants included those undergoing dialysis (1 study) [56], those receiving HD (15 studies) [33,41,43,45-48,50,52-55,58,59], those undergoing PD (4 studies) [32,40,44,49], and those undergoing KT (3 studies) [42,51,57].

### Characteristics of DHIs

The 23 studies involved various forms of DHIs, including mobile apps, videos, telemedicine, social media, VR technology, multimedia, and text messaging. Of these, 8 studies utilized video [32-34,42,43,47,51,56], 5 used mobile apps [44,45,48,50,52], 4 employed VR [46,49,54,58], 2 incorporated multimedia [55,57] and telemedicine [40,41], and 1 study each used social media [59] and text messaging [53].

The interventions varied in approach and content. They were categorized into six groups: (1) Educational and self-management interventions, providing knowledge on dialysis, symptom management, dietary management, and more (n=11) [33,34,42,44,47,50,51,53,55,57,59]; (2) Exercise interventions using wearable devices and VR technology (n=4) [46,49,54,58]; (3) Medication management and disease follow-up systems [40]; (4) Psychological interventions, including mindfulness therapy, problem-solving therapy, cognitive behavioral therapy, and culturally tailored psychological interventions (n=4) [32,43,48,52]; (5) Entertainment therapy, including watching comedy films (n=2) [45,56]; and (6) Comprehensive interventions based on home telemedicine models (n=1) [41]. The interventions varied widely in dosage and duration, ranging from 4 days to 1 year. Detailed intervention characteristics are shown in Table 1.

### Outcome Measurement

#### Psychological Health

The outcomes related to psychological health included depression, anxiety, and stress levels. Nine studies reported the effects of DHI on depression, using commonly employed scales such as CES-D (Center for Epidemiologic Studies Depression Scale), BDI (Beck Depression Inventory), BDI-II (Beck Depression Inventory-II), PHQ-9 (Patient Health Questionnaire-9) [40,43,45,46,48,52,54,56,58]. Six studies assessed anxiety levels using the GAD-7 (Generalized Anxiety Disorder-7), BAI (Beck Anxiety Inventory), S-STAI (State-Trait Anxiety Inventory), and the Hamilton Anxiety Rating Scale [32,34,43,46,48,56]. One study used the PSS (Perceived Stress Scale) to measure participants’ perceived stress levels [32].

#### Self-Efficacy

Ten studies reported on the impact of DHI on self-efficacy, with nine studies providing extractable data for meta-analysis. Six different scales were used to assess self-efficacy. Three studies employed the GSES (Generalized Self-Efficacy Scale) [40,42,51], two studies used the CDSES (Chronic Disease Self-Efficacy Scale) [47,59], and the remaining four studies used the Self-Efficacy for Managing Chronic Disease [32], the 15-item Dietary Self-Efficacy Questionnaire [50], the PD Self-Efficacy Scale [44], and the Chinese General Self-Efficacy Scale [49].

#### Quality of Life

Fourteen studies reported on QoL, using eight different tools to measure this outcome. The tools included the KTQ-25 (Kidney Transplant Quality of Life 25-Item Questionnaire), KDQOL-SF (Kidney Disease Quality of Life Short Form), KDQOL-36 (Kidney Disease Quality of Life 36-Item Short Form), SF-36 (Kidney Disease Quality of Life Short Form), SF-12 (12-Item Short Form Health Survey), the 9-item Thai Health Status Assessment Instrument, EuroQOL 5D (EuroQol-5 Dimensions), and a Single-item Quality of Life scale. Five studies assessed both the Physical Component Summary and Mental Component Summary of QoL [41,42,48,51,58], three studies focused on the symptom dimension of QoL in patients with kidney disease [44,57,58], and two studies provided a comprehensive evaluation of eight general QoL domains (pain, physical functioning, physical role difficulty, emotional role difficulty, vitality, mental health, general health, and social functioning) [33,40].

#### Patient Engagement and Experience

In six studies, adherence to the intervention among patients with ESKD was assessed by the proportion of participants who completed the majority of the intervention content relative to the total intervention group. However, the definition of “completing the majority of the intervention content” varied across studies, such as watching at least 80% of the video content. Additionally, seven studies investigated the user experience of patients with ESKD, with two studies employing the technology acceptance model or self-designed questionnaires to assess user experience and satisfaction, while the remaining five studies utilized qualitative interviews to gain in-depth insights into the experiences of patients with ESKD.

#### Risk of Bias and Quality of Evidence

**Figure 2.**
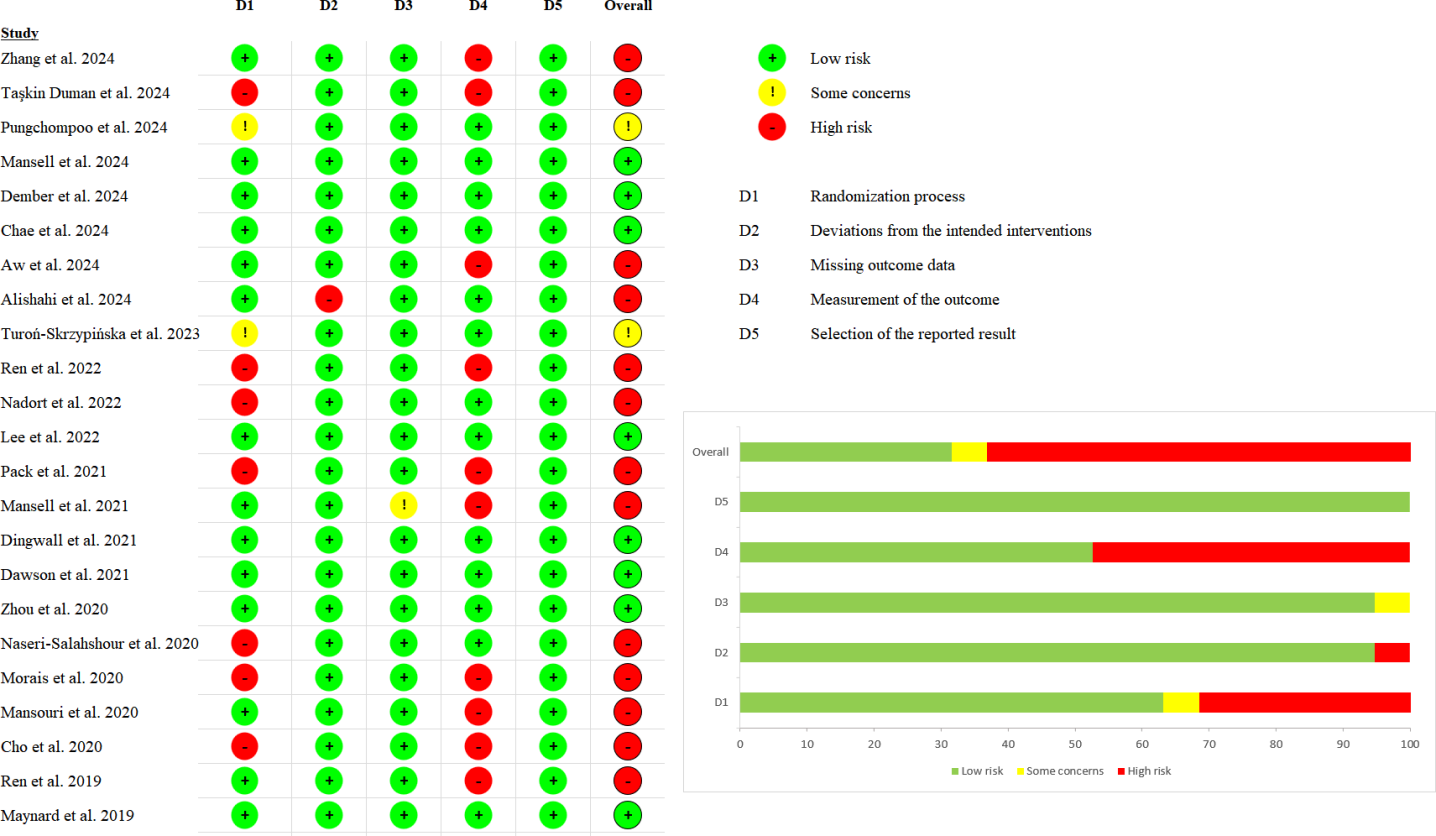
Risk of bias summary [32-34,40-59].

The results of the risk of bias assessment are shown in Figure 2. Eight studies were rated as “low risk” [42-44,49,52-54], 2 studies raised some concerns [41,46], and 13 studies were judged to be at “high risk” [32-34,40,45,47,48,50,51,55-57,59]. In Domain 1 (Randomization process), 7 studies had randomization based on the timing of dialysis treatments [33,34,47,48,50,55,56], leading to a high risk of bias in randomization, and 2 studies did not provide a detailed explanation of the randomization process [41,46], thus raising concerns about its methodology. In Domain 2 (Deviations from the intended interventions), one study used inappropriate analysis methods for estimating intervention effects [45]. In Domain 3 (Missing outcome data), one study had a high attrition rate without reporting reasons for dropout [51], raising concerns about potential bias. In Domain 4 (Measurement of the outcome), nine studies did not report whether outcome assessors were blinded [32,34,40,50,51,56,57,59], and one study used trained researchers to assess outcomes [47], resulting in a high risk of bias in outcome measurement for these ten studies. In Domain 5 (Selection of the reported result), all studies were rated as low risk.

**Figure 3.**
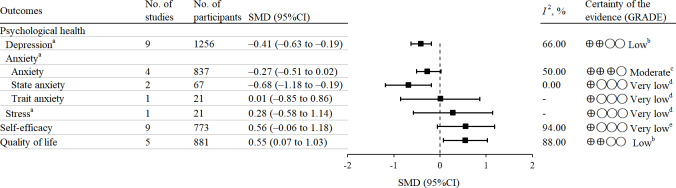
Meta-analysis summary of the included outcomes. (A) For meta-analysis on depression, anxiety, and stress, an effect size less than 0 indicates greater effectiveness of DHIs, while an effect size greater than 0 indicates greater effectiveness for other outcomes. (B) Downgraded by one level due to high risk of bias in included studies and by one level due to statistical heterogeneity. (C) Downgraded by one level due to high risk of bias in included studies. (D) Downgraded by one level due to high risk of bias in included studies and by two levels due to very serious imprecision due to small sample size and wide confidence intervals. (E) Downgraded by one level due to high risk of bias in included studies, by one level due to statistical heterogeneity, and by one level due to serious imprecision from wide confidence intervals.

The GRADE assessment of the quality of evidence for the included studies indicated that the evidence quality for depression and overall QoL was low and the evidence for general anxiety was moderate, while the evidence for stress and self-efficacy was very low. The downgrading of evidence quality was mainly attributed to bias risks in the included studies, inconsistency between studies, and imprecision of the results. The full assessment is provided in Figure 3, with additional details available in the Multimedia Appendix 2.

### Impact of DHIs on Primary Outcomes

#### Psychological Health

Nine studies evaluated the effectiveness of DHIs on depression levels in patients with ESKD. The pooled results showed that DHIs significantly improved depression levels (SMD −0.41, 95% CI −0.63 to −0.19, *P*=.003). Six studies assessed the effectiveness of DHIs on anxiety levels in patients with ESKD. DHIs significantly reduced both general anxiety (SMD −0.27, 95% CI −0.51 to −0.02, *P*=.03) and state anxiety (SMD −0.68, 95% CI −1.18 to −0.19, *P*=.007), while trait anxiety did not show significant improvement. Only one study explored the effect of DHI on perceived stress levels in patients with ESKD, and the results showed that, compared to the control group, there was no significant improvement in the intervention group’s perceived stress levels (Figure 3).

#### Self-Efficacy

Ten studies reported on the impact of DHIs on self-efficacy in patients with ESKD. Pooled analysis of nine studies revealed that DHIs did not significantly improve self-efficacy (SMD 0.56, 95% CI −0.06 to 1.18, *P*=.08; Figure 3).

#### Quality of Life

Five studies reported on the impact of DHIs on overall QoL in patients with ESKD, with pooled results showing a significant improvement (SMD 0.55, 95% CI 0.07-1.03, *P*=.03; Figure 3). Six studies separately reported the effects of DHIs on the Physical Component Summary and Mental Component Summary of QoL, and the pooled results indicated no significant improvement in either dimension (Multimedia Appendix 3, Figures 1.9-1.10). Three studies reported on the symptom dimension of QoL in patients with kidney disease. The pooled results showed that DHIs did not significantly improve the symptom dimension of QoL in patients with ESKD (Multimedia Appendix 3, Figure 1.8). Two studies conducted a comprehensive assessment of the eight general QoL dimensions, including pain, physical functioning, physical role difficulty, emotional role difficulty, mental health, and general health. The combined results from these studies indicated that DHIs significantly improved these dimensions of QoL in patients with ESKD (Multimedia Appendix 3, Figures 1.11-1.16). Additionally, one study was excluded from the meta-analysis due to a lack of data; however, its results also suggested that DHIs could improve the QoL in patients with ESKD [53].

### Patient Engagement and Experience Analysis

The included studies reported intervention completion rates exceeding 63%, with the majority demonstrating high completion rates (80%‐95%). Among studies evaluating adherence to DHIs in patients with ESKD, adherence rates ranged from 54% to 79%, with two studies reporting adherence rates above 75%. Furthermore, patients with ESKD generally provided positive feedback on DHIs, emphasizing perceived ease of use, safety, and utility.

### Subgroup Analysis

#### Treatment Modality

The meta-analysis compared the effects of DHIs on patients with ESKD undergoing different treatment modalities (PD, HD, and KT) ( Multimedia Appendix 3, Figure 2). The results showed that DHIs effectively improved depressive symptoms in patients receiving both PD and HD. However, DHIs had a more significant impact on depressive symptoms in patients receiving PD compared to those receiving HD. Since no studies examined the effect of DHIs on depression in recipients undergoing KT, this group was not included in the analysis. Additionally, DHIs improved self-efficacy and overall QoL in patients receiving HD.

#### Intervention Modality

We compared the effects of different DHI modalities (VR, video-based interventions, telemedicine, mobile apps, and social media) on depression, self-efficacy, and overall QoL in patients with ESKD ( Multimedia Appendix 3, Figure 3). The results indicated that VR, video-based interventions, and telemedicine had a significant positive impact on depressive symptoms in these patients. Mobile apps, telemedicine, and social media interventions were found to improve self-efficacy, while mobile apps and telemedicine also showed improvements in overall QoL.

#### Duration of Intervention

Subgroup analyses of different intervention durations for depression and overall QoL yielded contrasting results (Multimedia Appendix 3, Figure 4). For depression, interventions lasting 4‐8 weeks showed significantly greater improvements than those lasting longer than 8 weeks. For overall QoL, results showed that DHIs significantly improved QoL only when the intervention duration was at least 8 weeks. In terms of self-efficacy, due to the broad variation in intervention durations (ranging from 4 d to 1 y), we grouped studies based on whether the intervention lasted less than 3 months or 3 months and longer. The results indicated that DHIs with a duration of less than 3 months effectively improved self-efficacy in patients with ESKD.

#### Sensitivity Analysis

For depression, the overall effect of DHIs remained stable regardless of which study was excluded, indicating robust findings. In contrast, for general anxiety, statistical significance was observed only after removing the study by Nadort (SMD −0.33, 95% CI −0.51 to −0.14, *P*<.001) [48] (Multimedia Appendix 3, Figure 5.1). Regarding self-efficacy, the exclusion of individual studies generally did not alter the results, except for the study with the largest weight [43], whose removal rendered the effect statistically significant (SMD 0.29, 95% CI 0.02-0.57, *P*=.04), with a reduction in heterogeneity (*I*² dropped from 94% to 62%) (Multimedia Appendix 3, Figure 5.2). For overall QoL, the pooled effect became nonsignificant when the study with the largest weight was excluded [43] ( Multimedia Appendix 3, Figure 5.3).

## Discussion

### Principal Findings

This study demonstrated that DHIs significantly improved depressive symptoms, anxiety, and overall QoL in patients with ESKD compared to control groups. DHIs also substantially enhanced self-efficacy in patients receiving HD, particularly when interventions were delivered via mobile apps, telemedicine, or social media platforms. Video-based interventions proved effective in addressing both depressive symptoms and QoL. Additionally, patients with ESKD exhibited high engagement and positive experiences with DHIs, even among older adults with limited digital health literacy.

### Comparison to Prior Work

Existing evidence indicates that DHI can significantly reduce depression in patients with ESKD, which is consistent with similar findings in populations such as postpartum women [60], heart failure patients [61], and those with depression [62]. This effect may stem from the multidimensional nature of DHIs. In the studies included in our review, DHI strategies targeting depressive symptoms encompassed psychological support, exercise therapy, and recreational therapy. The digital format of these interventions overcame geographical barriers and improved access to care for patients with ESKD. Patients with ESKD often face fatigue [63], which reduces their willingness and adherence to exercise. Wearable devices and VR technologies can bridge this gap by providing remote monitoring and interactive exercise, enhancing engagement. Furthermore, DHIs demonstrated comparable efficacy in ameliorating generalized and state anxiety in patients with ESKD, with mechanisms mirroring those observed for depressive symptom relief. For instance, digital cognitive behavioral therapy guides patients to focus on the present, reduce disease-related uncertainty, and identify and address specific stressors, thereby strengthening problem-solving capabilities. Online educational interventions also empower patients with ESKD with disease knowledge and self-management skills, bolstering confidence in disease control and mitigating anxiety caused by information asymmetry. However, the majority of included studies focused on the impact of DHIs on anxiety in patients receiving HD, with limited attention to patients undergoing PD and KT, which may restrict the generalizability of the findings.

Subgroup analysis of depressive outcomes indicated that, apart from mobile apps, VR, video-based interventions, and telemedicine effectively alleviated depressive symptoms in patients with ESKD. This contrasts with the findings in other populations [60,62,64], likely due to limitations in intervention design, user characteristics, and cultural context in the included studies. One study required patients with ESKD to complete learning tasks on tablets within a short period, but the high dropout rate in the intervention group suggested that the complex design may not have adequately considered the needs of elderly patients. With an average participant age of 65 years [48], older patients typically prefer clear and simple user interfaces and instructions [30]. Complex interfaces and tasks may increase their anxiety and technical barriers, reducing intervention adherence and effectiveness. Another study targeting Indigenous Australian patients found that the app was only effective for those with moderate to severe depressive symptoms, possibly due to the “resilience and toughness” exhibited by Indigenous patients facing ESKD [52], which may weaken their response to interventions for mild depressive symptoms. Subgroup analysis also revealed that DHIs with a duration of 4‐8 weeks were more effective in improving depressive symptoms compared to interventions lasting more than 8 weeks. Short-term interventions typically have higher engagement and adherence rates, as patients are more likely to maintain motivation and focus, effectively absorbing and applying intervention content in daily life. Prolonged interventions may lead to reduced interest in the content, and long-term interventions may fail to adapt strategies to meet patients’ evolving needs, resulting in diminishing effects over time.

The meta-analysis results indicated that DHIs did not significantly improve overall self-efficacy in patients with ESKD, but subgroup analyses revealed other important results. While DHIs showed no statistically significant improvement in self-efficacy for patients undergoing PD and KT, they significantly enhanced self-efficacy in patients receiving HD, consistent with recent systematic reviews focusing on this subgroup [65]. This discrepancy may be due to baseline differences in self-efficacy levels among these groups. Patients on HD, who rely on dialysis centers, experience a heavier symptom burden, face limitations in social roles, and typically exhibit lower baseline self-efficacy, making them more responsive to DHIs that provide targeted support, real-time feedback, and educational resources. In contrast, patients undergoing PD and KT generally have higher baseline self-efficacy, as patients receiving PD manage their treatment more autonomously at home [66,67], and KT recipients experience significant improvements in QoL posttransplantation [68]. This higher baseline level may limit the potential for further self-efficacy enhancement through DHIs, particularly when interventions fail to address their more complex needs (eg, operational skills for patients receiving or medication adherence for transplant recipients). Additionally, DHIs delivered via mobile apps, telemedicine, and social media offer advantages such as real-time feedback, personalized guidance, and interactive features, providing emotional support and enhancing patients’ sense of control and confidence in disease management. Compared to video-based interventions, which require passive information reception, these interactive DHI formats significantly improve self-efficacy in patients with ESKD [69].

DHIs also effectively improved the QoL in patients with ESKD, with a greater effect size compared to their application in other chronic disease populations [70], such as cancer survivors [71,72] and patients having HIV [73]. The QoL of patients with ESKD is significantly impacted by severe symptom burdens (eg, fatigue, pain, fluid overload), complex medication and dialysis regimens, and poor mental health [7,74]. However, patients with ESKD often exhibit low health literacy, which diminishes their ability to engage in effective self-management, thereby amplifying their need for structured and accessible interventions [75,76]. The treatment and self-management needs of patients with ESKD are relatively clear and focused, allowing DHIs to provide highly targeted disease management tools and multidimensional health education. These interventions help patients with ESKD master self-management techniques for disease symptoms, enhance coping abilities, and improve mental health, thereby effectively enhancing their QoL. In contrast, the QoL of cancer survivors varies widely due to the diversity of cancer types, varying malignancy levels, and heterogeneous self-management needs, making it challenging for DHIs to comprehensively address their diverse requirements. Similarly, the QoL of patients having HIV is heavily influenced by complex psychosocial factors such as stigma and social discrimination, which may fall outside the scope of existing DHIs [73].

Subgroup analysis revealed that mobile apps and educational videos are effective DHI formats for improving QoL in patients with ESKD, consistent with findings from a systematic review in patients with chronic obstructive pulmonary disease [77]. Although only one included study utilized a mobile app, it demonstrated the potential of highly targeted, interactive apps in enhancing QoL. This app assisted patients in dietary self-management by providing real-time feedback on biochemical indicators in food, identifying dietary issues, and helping control intake, thereby improving self-efficacy and QoL [50]. Video-based education, on the other hand, effectively conveyed disease knowledge and self-management skills through intuitive and engaging formats, enhancing health literacy and self-management capabilities. Both DHI formats offer accessible and flexible support, particularly in resource-limited settings or for patients with mobility challenges, significantly improving intervention accessibility and adherence. Furthermore, intervention duration is a critical factor influencing the effectiveness of DHIs in improving QoL. Subgroup analysis indicated that interventions lasting ≥8 weeks were necessary to achieve significant improvements, likely due to the complexity and multidimensional nature of QoL. QoL encompasses physical function, mental health, social function, and disease burden [78], and improvements in these areas require sustained intervention over time. As shown in our meta-analysis, not all QoL dimensions showed significant improvement after DHI implementation. Longer intervention durations provide patients with ESKD with sufficient time to gradually acquire disease management skills, establish healthy behavioral habits, and enhance self-efficacy and psychological resilience through ongoing health education and support, ultimately leading to improved QoL.

Patients with ESKD demonstrated high overall acceptance of DHIs, with a marked preference for interactive designs and digital educational content. Notably, gamified exercise interventions utilizing VR and real-time feedback mechanisms effectively enhanced patient engagement. Furthermore, video-based tailored health education achieved exceptionally high satisfaction rates, highlighting patients’ simultaneous demand for both engaging formats and practical utility in digital content. However, the persistent digital divide, manifested through operational barriers related to tablet interfaces, app navigation, and VR systems, remains a critical barrier to adherence, particularly given the predominance of older adults in the ESKD population.

Overall, these findings indicate that DHIs provide practical and scalable solutions to address the complex psychosocial needs, self-management challenges, and quality-of-life issues faced by patients with ESKD. DHIs can both complement routine clinical care and reduce dependence on in-person consultations, while enhancing continuity of support between dialysis sessions. Importantly, their flexibility allows for adaptation to individual patient preferences and resource constraints, enabling health care providers to deliver precise, patient-centered care.

### Strengths and Limitations

This systematic review has several strengths. First, we strictly followed predefined inclusion and exclusion criteria to ensure the scientific rigor and reliability of the studies. Second, we selected studies published between 2019 and 2024, reflecting the most recent advancements in the field of DHI. Third, the studies included in this review were from countries with varying income levels, enhancing the global representativeness of the results. Finally, sensitivity and subgroup analyses were conducted to validate the stability of the results and explore potential sources of heterogeneity. However, there are several limitations to this review. First, we only included studies published in English, which may have resulted in the omission of important research in other languages. Second, several of the included studies had a high risk of bias, and some results exhibited significant heterogeneity, leading to a lower quality of evidence. Therefore, the findings should be interpreted with caution. Additionally, due to the lack of consistent and comprehensive follow-up data, this review only assessed the immediate effects of the interventions and did not evaluate the long-term impact of DHIs.

### Implications for Future Research

Further research is needed to confirm the impact of DHI on self-efficacy, particularly in patients undergoing PD and KT. Interactive, personalized interventions, such as telemedicine, apps, and intuitive video-based education, show significant potential for improving patient outcomes. These forms of DHI should be considered for integration into routine health education and follow-up management for patients with ESKD.

The COVID-19 pandemic has accelerated the development and adoption of DHI, but it has also highlighted many limitations in DHIs introduced post-pandemic [29]. As shown in this review, the quality of current research on DHI in the areas of mental health, self-efficacy, and QoL for patients with ESKD is generally low. To better integrate digital health into current health care systems, future research should focus on the following aspects to enhance and expand the application of DHI for patients with ESKD. First, study designs should be optimized, with more high-quality RCTs conducted to improve evidence quality. Follow-up durations should be extended to evaluate the long-term effects of DHI, and the scope of research should be broadened to include underrepresented groups, such as patients undergoing PD and KT. Besides, the design of DHIs should leverage the strengths of digital health, personalization, interactivity, and user engagement by tailoring interventions to the specific needs and characteristics of different populations. For example, older patients, an important subgroup of patients with ESKD, often face limitations in the usability of many self-care apps due to complex interfaces, operational difficulty, and poor content adaptability. Future research should balance innovative features with age-friendly design by prioritizing the development of DHIs featuring simplified operational workflows, integrated voice assistants, and intuitive culturally adapted interfaces to enhance acceptance rates and intervention efficacy among older patients with ESKD.

### Conclusion

Our systematic review suggests that DHIs can significantly alleviate depression and anxiety, improve the QoL in patients with ESKD, and have a positive impact on self-efficacy in patients receiving HD. Mobile apps, telemedicine, and video-based interventions show promise as effective DHI formats for improving the physical and mental health of patients with ESKD. Intervention duration played a critical role: shorter interventions (4‐8 wk) were more effective in alleviating depressive symptoms, whereas longer interventions (≥8 wk) were necessary to significantly improve overall QoL. However, the overall quality of the evidence is low, and caution should be exercised when applying these findings. Future research should adopt more rigorous designs and extend follow-up periods. DHIs should focus more on personalization and interactivity, particularly optimizing them for older patients, a key demographic in ESKD care. Additionally, patients undergoing PD and KT have not been sufficiently explored in DHI research, and their treatment models and psychosocial needs differ significantly from those of patients receiving HD. Future studies should investigate the impact of DHI in these populations.

## Supplementary material

10.2196/74414 Multimedia Appendix 1 The search strategies.

10.2196/74414 Multimedia Appendix 2 GRADE evidence profile.

10.2196/74414 Multimedia Appendix 3 Forest plot.

10.2196/74414 Checklist 1 PRISMA checklist.

## Abbreviations

BAI: Beck Anxiety Inventory
BDI: Beck Depression Inventory
BDI-II: Beck Depression Inventory-II
CDSES: Chronic Disease Self-Efficacy Scale
CES-D: Center for Epidemiologic Studies Depression Scale
CIs: confidence intervals
DHIs: digital health interventions
ESKD: end-stage chronic kidney disease
EuroQol-5D: EuroQol-5 Dimensions
GAD-7: Generalized Anxiety Disorder-7
GRADE: grading of recommendation, assessment, development, and evaluation
GSES: Generalized Self-Efficacy Scale
HD: hemodialysis
KDQOL-36: Kidney Disease Quality of Life 36-Item Short Form
KDQOL-SF: Kidney Disease Quality of Life Short Form
KT: kidney transplantation
KTQ-25: Kidney Transplant Quality of Life 25-Item Questionnaire
PD: peritoneal dialysis
PHQ-9: Patient Health Questionnaire-9
PRISMA: Preferred Reporting Items for Systematic Reviews and Meta-Analyses
PSS: Perceived Stress Scale
QoL: quality of life
RCT: randomized controlled trial
RoB2: risk of bias tool 2
S-STAI: State-Trait Anxiety Inventory
SF-12: 12-Item Short Form Health Survey
SF-36: Kidney Disease Quality of Life Short Form
SMD: standardized mean differences
VR: virtual reality
