# Supplementary material for: The Impact of Digital Health Interventions on Psychological Health, Self-Efficacy, and Quality of Life in Patients With End-Stage Kidney Disease: Systematic Review and Meta-Analysis
Source: J Med Internet Res. 2025 Sep 26;27:e74414. doi: 10.2196/74414 (PMC12466795; doi:10.2196/74414)
Supplement: Multimedia Appendix 1 [file jmir-v27-e74414-s001.doc]

| Database | Search Strategies | Results |
| --- | --- | --- |
| PubMed | #1 (kidney failure, chronic[MeSH Terms]) OR (chronic kidney failure[Text Word]) OR (kidney failure, chronic[Text Word]) OR (renal failure, chronic[Text Word]) OR (chronic renal failure[Text Word]) OR (end stage renal disease[Text Word]) OR (end stage kidney disease[Text Word]) OR (uremia[MeSH Terms]) OR (uremia*[Text Word]) OR (uraemia[Text Word]) OR (renal dialysis[MeSH Terms]) OR (renal dialysis[Text Word]) OR (kidney dialysis[Text Word]) OR (kidney transplantation[MeSH Terms]) OR (kidney transplantation[Text Word]) OR (renal transplantation[Text Word]) | 326195 |
|  | #2 (mental health[MeSH Terms]) OR (mental health[Text Word]) OR (psychological health[Text Word]) | 317445 |
|  | #3 (depression[MeSH Terms]) OR (depression*[Text Word]) OR (depressive disorder[MeSH Terms]) OR (depressive disorder[Text Word]) OR (depressed[Text Word]) OR (depressive*[Text Word]) | 666566 |
|  | #4 (anxiety[MeSH Terms]) OR (anxiet*[Text Word]) | 350979 |
|  | #5 (stress*[Text Word]) | 1392541 |
|  | #6 #2 OR #3 OR #4 OR #5 | 2295592 |
|  | #7 (self efficacy[MeSH Terms]) OR (self efficacy[Text Word]) | 55491 |
|  | #8 (quality of life[MeSH Terms]) OR (quality of life[Text Word]) OR (life quality[Text Word]) OR (health related quality of life[Text Word]) OR (HRQOL[Text Word]) OR (QoL[Text Word]) | 499089 |
|  | #9 #6 OR #7 OR #8 | 2721734 |
|  | #10 (digital health[MeSH Terms]) OR (digital health[Text Word]) OR (telemedicine[MeSH Terms]) OR (telemedicine[Text Word]) OR (mobile health[Text Word]) OR (mHealth[Text Word]) OR (telehealth[Text Word]) OR (eHealth[Text Word]) OR (internet-based intervention[MeSH Terms]) OR (internet based intervention[Text Word]) OR (online intervention[Text Word]) OR (mobile applications[MeSH Terms]) OR (mobile application*[Text Word]) OR (smartphone[MeSH Terms]) OR (smartphone[Text Word]) OR (telephone[MeSH Terms]) OR (telephone[Text Word]) OR (phon*[Text Word]) OR (social media[MeSH Terms]) OR (social media[Text Word]) OR (technology[MeSH Terms]) OR (technolog*[Text Word]) OR (telehealth*[Text Word]) OR (videotape recording[MeSH Terms]) OR (videotape recording[Text Word]) OR (video*[Text Word]) OR (blogging[MeSH Terms]) OR (blog*[Text Word]) OR (computers[MeSH Terms]) OR (comput*[Text Word]) OR (remote consultation[MeSH Terms]) OR (remote consultation[Text Word]) OR (electronics[MeSH Terms]) OR (electronic*[Text Word]) | 3939342 |
|  | #11 "Clinical Trial"[Publication Type] OR "Controlled Clinical Trial"[Publication Type] OR "Randomized Controlled Trial"[Publication Type] OR "Controlled Clinical Trials as Topic"[MeSH Terms] OR "Random Allocation"[MeSH Terms] OR "Double-Blind Method"[MeSH Terms] OR "Single-Blind Method"[MeSH Terms] OR "randomized controlled trial*"[Text Word] OR "RCT"[Text Word] OR "random*"[Text Word] OR "trial"[Text Word] OR "clinical trial*"[Text Word] OR "single blind*"[Text Word] OR "double blind*"[Text Word] OR "triple blind*"[Text Word] | 2802607 |
|  | #12 #1 AND #9 AND #10 AND #11 | 375 |
| Web of Science | #1 TS=(kidney failure, chronic OR chronic kidney failure OR renal failure, chronic OR chronic renal failure OR end stage renal disease OR end stage kidney disease OR uremia OR renal dialysis OR kidney dialysis OR kidney transplantation OR renal transplantation) | 563623 |
|  | #2 TS=(mental health OR psychological health OR depression OR depressive disorder OR depressed OR depressive OR anxiety OR stress OR self efficacy OR quality of life OR quality of life OR health related quality of life OR HRQOL OR QoL) | 7771011 |
|  | #3 TS=(digital health OR telemedicine OR mobile health OR mealth OR telehealth OR eHealth OR internet-based intervention OR online intervention OR mobile applications OR smartphone OR telephone OR social media OR technology OR telehealth OR videotape recording OR video OR blogging OR blog OR computer OR remote consultation OR electronics) | 18387619 |
|  | #4 TS=(“randomized controlled trial*” OR “RCT” OR “random*” OR “trial” OR “clinicaltrial*” OR “single blind*” OR “double blind*” OR “triple blind*”) | 4839569 |
|  | #5 #1 AND #2 AND #3 AND #4 | 1539 |
| Cochrane library | #1 MeSH descriptor: [kidney failure, chronic] explode all trees | 6103 |
|  | #2 MeSH descriptor: [Uremia] explode all trees | 6158 |
|  | #3 MeSH descriptor: [Renal Dialysis] explode all trees | 7520 |
|  | #4 MeSH descriptor: [Kidney Transplantation] explode all trees | 4732 |
|  | #5 (kidney failure, chronic):ti,ab,kw OR (chronic kidney failure):ti,ab,kw OR (chronic renal failure):ti,ab,kw OR (renal failure, chronic):ti,ab,kw OR (end stage renal disease):ti,ab,kw | 20934 |
|  | #6 (end stage kidney disease):ti,ab,kw OR (uremia):ti,ab,kw OR (uraemia):ti,ab,kw OR (renal dialysis):ti,ab,kw OR (kidney dialysis):ti,ab,kw | 18722 |
|  | #7 (kidney transplantation):ti,ab,kw AND (renal transplantation):ti,ab,kw | 7014 |
|  | #8 #1 OR #2 OR #3 OR #4 OR #5 OR #6 OR #7 | 35744 |
|  | #9 MeSH descriptor: [Mental Health] explode all trees | 3462 |
|  | #10 MeSH descriptor: [Depression] explode all trees | 19070 |
|  | #11 MeSH descriptor: [Depressive Disorder] explode all trees | 17024 |
|  | #12 MeSH descriptor: [Anxiety] explode all trees | 13320 |
|  | #13 (mental health):ti,ab,kw OR (psychological health):ti,ab,kw OR (depress*):ti,ab,kw OR (depressive disorder):ti,ab,kw OR (anxiety):ti,ab,kw | 201336 |
|  | #14 (anxiet*):ti,ab,kw OR (stress*):ti,ab,kw | 156092 |
|  | #15 #9 OR #10 OR #11 OR #12 OR #13 OR #14 | 260050 |
|  | #16 MeSH descriptor: [Self Efficacy] explode all trees | 4568 |
|  | #17 MeSH descriptor: [Quality of Life] explode all trees | 45881 |
|  | #18 (self efficacy):ti,ab,kw OR (quality of life):ti,ab,kw OR (life quality):ti,ab,kw OR (health related quality of life):ti,ab,kw OR (HRQOL):ti,ab,kw | 219306 |
|  | #19 (QoL):ti,ab,kw | 30891 |
|  | #20 #16 OR #17 OR #18 | 219338 |
|  | #21 MeSH descriptor: [Digital Health] explode all trees | 28 |
|  | #22 MeSH descriptor: [Telemedicine] explode all trees | 5219 |
|  | #23 MeSH descriptor: [Internet-Based Intervention] explode all trees | 816 |
|  | #24 MeSH descriptor: [Mobile Applications] explode all trees | 2234 |
|  | #25 MeSH descriptor: [Smartphone] explode all trees | 1259 |
|  | #26 MeSH descriptor: [Telephone] explode all trees | 6517 |
|  | #27 MeSH descriptor: [Social Media] explode all trees | 627 |
|  | #28 MeSH descriptor: [Technology] explode all trees | 9416 |
|  | #29 MeSH descriptor: [Videotape Recording] explode all trees | 1117 |
|  | #30 MeSH descriptor: [Blogging] in all MeSH products | 25 |
|  | #31 MeSH descriptor: [Computers] explode all trees | 3127 |
|  | #32 MeSH descriptor: [Remote Consultation] explode all trees | 486 |
|  | #33 MeSH descriptor: [Electronics] in all MeSH products | 1677 |
|  | #34 (digital health):ti,ab,kw OR (telemedicine):ti,ab,kw OR (mobile health):ti,ab,kw OR (mHealth):ti,ab,kw OR (telehealth):ti,ab,kw | 26341 |
|  | #35 (eHealth):ti,ab,kw OR (internet-based intervention):ti,ab,kw OR (online intervention):ti,ab,kw OR (mobile application*):ti,ab,kw OR (smartphone):ti,ab,kw | 33373 |
|  | #36 (telephone):ti,ab,kw OR (phon*):ti,ab,kw OR (social media):ti,ab,kw OR (telehealth*):ti,ab,kw OR (technolog*):ti,ab,kw | 88099 |
|  | #37 (videotape recording):ti,ab,kw OR (video*):ti,ab,kw OR (blog*):ti,ab,kw OR (comput*):ti,ab,kw OR (remote consultation):ti,ab,kw | 124373 |
|  | #38 (electronic*):ti,ab,kw | 26158 |
|  | #39 #29 OR #30 OR #31 OR #32 OR #33 OR #34 OR #35 OR #36 OR #37 OR #38 | 242007 |
|  | #40 #8 AND #15 #20 AND #39 | 227 |
| Embase | #1 'chronic kidney failure'/exp | 235658 |
|  | #2 'end stage renal disease'/exp | 61200 |
|  | #3 'uremia'/exp | 33283 |
|  | #4 'hemodialysis'/exp | 153576 |
|  | #5 'peritoneal dialysis'/exp | 54775 |
|  | #6 'kidney transplantation'/exp | 200965 |
|  | #7 'chronic kidney disease':ab,ti OR 'chronic kidney disorder':ab,ti OR 'chronic kidney insufficiency':ab,ti OR 'chronic nephropathy':ab,ti OR 'chronic renal disease':ab,ti OR 'chronic renal failure':ab,ti OR 'chronic renal insufficiency':ab,ti OR 'kidney chronic failure':ab,ti OR 'kidney disease, chronic':ab,ti OR 'kidney failure, chronic':ab,ti OR 'kidney function, chronic disease':ab,ti OR 'renal insufficiency, chronic':ab,ti OR 'chronic kidney failure':ab,ti OR 'end stage kidney disease':ab,ti OR 'end stage kidney failure':ab,ti OR 'end stage renal dysfunction':ab,ti OR 'end stage renal failure':ab,ti OR 'end stage renal impairment':ab,ti OR 'end stage renal insufficiency':ab,ti OR 'end-stage kidney disease':ab,ti OR 'end-stage kidney failure':ab,ti OR 'end-stage renal disease':ab,ti OR 'esrd':ab,ti OR 'stage 5 kidney disease':ab,ti OR 'stage 5 renal disease':ab,ti OR 'end stage renal disease':ab,ti OR 'azotaemia':ab,ti OR 'azotemia':ab,ti OR 'hyperazotemia':ab,ti OR 'hyperuraemia':ab,ti OR 'hyperuremia':ab,ti OR 'uraemia':ab,ti OR 'uraemic serum':ab,ti OR 'uraemic syndrome':ab,ti OR 'uremic serum':ab,ti OR 'uremic syndrome':ab,ti OR 'uremia':ab,ti OR 'blood dialysis':ab,ti OR 'dialysis center':ab,ti OR 'dialysis, blood':ab,ti OR 'extracorporeal blood cleansing':ab,ti OR 'extracorporeal dialysis':ab,ti OR 'haemodialysis':ab,ti OR 'haemodialysis center':ab,ti OR 'haemodialysis centre':ab,ti OR 'haemodialysis department':ab,ti OR 'haemodialysis unit':ab,ti OR 'haemodialysis units, hospital':ab,ti OR 'hemodialyse':ab,ti OR 'hemodialysis center':ab,ti OR 'hemodialysis department':ab,ti OR 'hemodialysis unit':ab,ti OR 'hemodialysis units, hospital':ab,ti OR 'hemorenodialysis':ab,ti OR 'hemotrialysate':ab,ti OR 'hospital haemodialysis units':ab,ti OR 'hospital hemodialysis units':ab,ti OR 'renal dialysis':ab,ti OR 'hemodialysis':ab,ti OR 'dialysis, peritoneal':ab,ti OR 'peritoneum dialysis':ab,ti OR 'peritoneal dialysis':ab,ti OR 'kidney allograft transplantation':ab,ti OR 'kidney allotransplantation':ab,ti OR 'kidney cadaver transplantation':ab,ti OR 'kidney grafting':ab,ti OR 'kidney homotransplantation':ab,ti OR 'kidney retransplantation':ab,ti OR 'renal homotransplantation':ab,ti OR 'renal transplantation':ab,ti OR 'second set kidney transplantation':ab,ti OR 'transplantation, kidney':ab,ti OR 'kidney transplantation':ab,ti | 461315 |
|  | #8 #1 OR #2 OR #3 OR #4 OR #5 OR #6 OR #7 | 674045 |
|  | #9 'mental health'/exp | 281696 |
|  | #10 'depression'/exp | 717878 |
|  | #11 'anxiety'/exp | 340552 |
|  | #12 'physiological stress'/exp | 549568 |
|  | #13 ('mental health' OR 'condition, mental' OR 'health, mental' OR 'mental care' OR 'mental condition' OR 'mental factor' OR 'mental help' OR 'mental service' OR 'mental state' OR 'mental status' OR 'mental status schedule' OR 'psychic health' OR 'mental health'):ab,ti OR (('depression' OR 'central depression' OR 'clinical depression' OR 'depressive disease' OR 'depressive disorder' OR 'depressive episode' OR 'depressive illness' OR 'depressive personality disorder' OR 'depressive state' OR 'depressive symptom' OR 'depressive syndrome' OR 'mental depression' OR 'parental depression' OR 'depression'):ab,ti) OR (('anxiety'):ab,ti) OR (('physiological stress' OR 'alarm reaction' OR 'biologic stress' OR 'biological stress' OR 'organismal stress' OR 'physiologic stress' OR 'physiological stresses' OR 'stress' OR 'stress capacity' OR 'stress reaction' OR 'stress resistance' OR 'stress response' OR 'stress situation' OR 'stress tolerance' OR 'stress, physiological' OR 'physiological stress'):ab,ti) | 2297279 |
|  | #14 #9 OR #10 OR #11 OR #12 OR #13 | 2806070 |
|  | #15 'self concept'/exp | 273856 |
|  | #16 'quality of life'/exp | 736402 |
|  | #17 ('self concept' OR 'concept, self' OR 'self' OR 'self attitude' OR 'self awareness' OR 'self confrontation' OR 'self efficacy' OR 'self image' OR 'self perception' OR 'self rating' OR 'self representation' OR 'self concept' OR 'self concept'):ab,ti OR (('quality of life' OR 'health related quality of life' OR 'HRQL' OR 'life quality' OR 'quality of life'):ab,ti) | 1869903 |
|  | #18 #15 OR #16 OR #17 | 2221130 |
|  | #19 'digital health'/exp | 2756 |
|  | #20 'telemedicine'/exp | 81773 |
|  | #21 'web-based intervention'/exp | 3822 |
|  | #22 'mobile application'/exp | 31166 |
|  | #23 'smartphone'/exp | 32615 |
|  | #24 'telephone'/exp | 47538 |
|  | #25 'social media'/exp | 61339 |
|  | #26 'technology'/exp | 291592 |
|  | #27 'videorecording'/exp | 130781 |
|  | #28 'blogging'/exp | 1062 |
|  | #29 'computer'/exp | 188352 |
|  | #30 'teleconsultation'/exp | 17772 |
|  | #31 'electronics'/exp | 91510 |
|  | #32 ('digital health' OR 'tele medicine' OR 'virtual medicine' OR 'telemedicine' OR 'telemedicine'):ab,ti OR (('web-based intervention' OR 'internet-based intervention' OR 'internet-intervention' OR 'online-based intervention' OR 'online-intervention' OR 'web intervention' OR 'web-based intervention'):ab,ti) OR (('mobile app' OR 'mobile applications' OR 'mobile apps' OR 'portable software app' OR 'portable software application' OR 'portable software applications' OR 'portable software apps' OR 'tablet application' OR 'mobile application' OR 'mobile app' OR 'mobile applications' OR 'mobile apps' OR 'portable software app' OR 'portable software application' OR 'portable software applications' OR 'portable software apps' OR 'tablet application' OR 'mobile application'):ab,ti) OR (('smartphone' OR 'smart phone' OR 'smartphones' OR 'smartphone' OR 'dataphone' OR 'telephone line' OR 'telephone'):ab,ti) OR (('Facebook' OR 'Flickr' OR 'hashtag' OR 'Instagram' OR 'LinkedIn' OR 'MySpace' OR 'Pinterest' OR 'Reddit' OR 'Sina Weibo' OR 'Snapchat' OR 'social networking platform' OR 'social networking site' OR 'social networking website' OR 'social platform' OR 'TikTok' OR 'Tumblr' OR 'Twitter' OR 'WeChat' OR 'WhatsApp' OR 'YouTube' OR 'social media' OR 'telephone'):ab,ti) OR (('social media' OR 'high technology' OR 'technician trainee' OR 'technological society' OR 'technologics' OR 'technology transfer' OR 'technology'):ab,ti) OR (('ampex' OR 'recording, video' OR 'video' OR 'video disc recording' OR 'video recording' OR 'video storage' OR 'video tape recording' OR 'video-taping' OR 'videodisc recording' OR 'videotape recording' OR 'videotaping' OR 'videorecording' OR 'technology'):ab,ti) OR (('videorecording' OR 'blog' OR 'blogs' OR 'blogging' OR 'computer environment' OR 'computer peripherals' OR 'computer science' OR 'computers' OR 'hardware' OR 'computer'):ab,ti) OR (('long distance consultation' OR 'remote consultation' OR 'tele-consultation' OR 'telephone consultation' OR 'telephone-based consultation' OR 'teleconsultation' OR 'electronics'):ab,ti) | 1399019 |
|  | #33 #19 OR #20 OR #21 OR #22 OR #23 OR #24 OR #25 OR #26 OR #27 OR #28 OR #29 OR #30 OR #31 OR #32 | 1812396 |
|  | #34 'clinical trial'/de OR 'controlled clinical trial'/de OR 'randomized controlledtrial' OR 'controlled clinical trial (topic)'/exp OR 'random allocation'/exp OR 'double-blind method'/exp OR 'single blind procedure'/exp | 1694083 |
|  | #35 'randomized controlled trial*':ab,ti OR 'rct':ab,ti OR 'random*':ab,ti OR 'trial':ab,ti OR 'clinical trial*':ab,ti OR 'single blind*':ab,ti OR 'double blind*':ab,ti OR 'triple blind*':ab,ti | 3107704 |
|  | #36 #34 OR #35 | 3862071 |
|  | #35 #8 AND (#14 OR #18) AND #33 AND #36 | 702 |
| CINAHL  (EBSCO) | S1 (MH "Kidney Failure, Chronic") OR (MH "Dialysis") OR (MH "Dialysis Patients") OR (MH "Peritoneal Dialysis") OR (MH "Dialysis Technicians") OR (MH "Hemodialysis") OR (MH "Uremia") OR (MH "Kidney Transplantation")  Expanders - Apply equivalent subjects  Search modes - Find all my search terms | 53717 |
|  | S2 SU (chronic kidney failure OR kidney failure, chronic OR renal failure, chronic OR chronic renal failure OR end stage renal disease OR end stage kidney disease OR uremia* OR uraemia OR dialysis OR dialysis Patients OR dialysis technicians OR renal dialysis OR kidney dialysis OR peritoneal Dialysis OR hemodialysis OR kidney transplantation OR renal transplantation)  Expanders - Apply equivalent subjects  Search modes - Find all my search terms | 57712 |
|  | S3 S1 OR S2  Expanders - Apply equivalent subjects  Search modes - Find all my search terms | 57712 |
|  | S4 (MH "Mental Health") OR (MH "Depression") OR (MH "Anxiety") OR (MH "Stress, Psychological")  Expanders - Apply equivalent subjects  Search modes - Find all my search terms | 265078 |
|  | S5 SU (mental health OR psychological health OR depression* OR depressive* OR depressive disorder OR depressed OR anxiet* OR stress*)  Expanders - Apply equivalent subjects  Search modes - Find all my search terms | 475766 |
|  | S6 S4 OR S5  Expanders - Apply equivalent subjects  Search modes - Find all my search terms | 475766 |
|  | S7 (MH "Self-Efficacy")  Expanders - Apply equivalent subjects  Search modes - Find all my search terms | 28629 |
|  | S8 (MH "Quality of Life")  Expanders - Apply equivalent subjects  Search modes - Find all my search terms | 150484 |
|  | S9 SU (quality of life OR life quality OR health related quality of life OR HRQOL OR QoL)  Expanders - Apply equivalent subjects  Search modes - Find all my search terms | 191811 |
|  | S10 S8 OR S9  Expanders - Apply equivalent subjects  Search modes - Find all my search terms | 176692 |
|  | S11 S6 OR S7 OR S10  Expanders - Apply equivalent subjects  Search modes - Find all my search terms | 633566 |
|  | S12 (MH "Digital Health") OR (MH "Digital Technology")  OR (MH "Telemedicine") OR (MH "Telehealth")  OR (MH "Internet-Based Intervention")  OR (MH "Mobile Applications") OR (MH "Smartphone") OR (MH "Text Messaging")  OR (MH "Telephone")  OR (MH "Social Media")  OR (MH "Technology")  OR (MH "Videorecording")  OR (MH "Blogs") OR (MH "Computers, Portable") OR (MH "Remote Consultation") OR (MH "Electronics")  Expanders - Apply equivalent subjects  Search modes - Find all my search terms | 153150 |
|  | S13 SU (digital health OR telemedicine OR mobile health OR mHealth OR telehealth OR eHealth OR internet-based intervention OR online intervention OR mobile application* OR smartphone OR telephone OR phon* OR social media OR technolog* OR telehealth* OR Videorecording OR videotape recording OR video* OR blog* OR comput* OR remote consultation OR electronic*)  Expanders - Apply equivalent subjects  Search modes - Find all my search terms | 539857 |
|  | S14 S12 OR S13  Expanders - Apply equivalent subjects  Search modes - Find all my search terms | 768347 |
|  | S15 S3 AND S11 AND S14  Expanders - Apply equivalent subjects  Search modes - Find all my search terms | 86 |
| PsycINFO  (Ovid) | S1 MA (kidney failure, chronic OR uremia OR dialysis OR Dialysis Patients OR Peritoneal Dialysis OR Hemodialysis OR Dialysis Technicians OR kidney transplantation)  Expanders - Apply equivalent subjects  Search modes - Find all my search terms | 2199 |
|  | S2 SU (chronic kidney failure OR kidney failure, chronic OR renal failure, chronic OR chronic renal failure OR end stage renal disease OR end stage kidney disease OR uremia* OR uraemia OR renal dialysis OR kidney dialysis OR dialysis OR Dialysis Patients OR Peritoneal Dialysis OR Hemodialysis OR Dialysis Technicians OR kidney transplantation OR renal transplantation)  Expanders - Apply equivalent subjects  Search modes - Find all my search terms | 4324 |
|  | S3 S1 OR S2  Expanders - Apply equivalent subjects  Search modes - Find all my search terms | 4324 |
|  | S4 MA (Mental Health OR Depression OR Anxiety OR Stress, Psychological OR Self-Efficacy OR Quality of Life)  Expanders - Apply equivalent subjects  Search modes - Find all my search terms | 230974 |
|  | S5 SU (mental health OR psychological health OR depression* OR depressive* OR depressive disorder OR depressed OR anxiet* OR stress* OR Self-Efficacy OR quality of life OR life quality OR health related quality of life OR HRQOL OR QoL)  Expanders - Apply equivalent subjects  Search modes - Find all my search terms | 837314 |
|  | S6 S4 OR S5  Expanders - Apply equivalent subjects  Search modes - Find all my search terms | 837314 |
|  | S7 MA (Digital Health OR Digital Technology OR Telemedicine OR Telehealth OR Internet-Based Intervention OR Mobile Applications OR Smartphone OR Text Messaging OR elephone OR Social Media OR Technology OR Videorecording OR Blogs OR Computers, Portable OR Remote Consultation OR Electronics)  Expanders - Apply equivalent subjects  Search modes - Find all my search terms | 16003 |
|  | S8 SU (digital health OR telemedicine OR mobile health OR mHealth OR telehealth OR eHealth OR internet-based intervention OR online intervention OR mobile application* OR smartphone OR telephone OR phon* OR social media OR technolog* OR telehealth* OR Videorecording OR videotape recording OR video* OR blog* OR comput* OR remote consultation OR electronic*)  Expanders - Apply equivalent subjects  Search modes - Find all my search terms | 363682 |
|  | S9 S7 OR S8  Expanders - Apply equivalent subjects  Search modes - Find all my search terms | 363907 |
|  | S10 S3 AND S6 AND S9  Expanders - Apply equivalent subjects  Search modes - Find all my search terms | 34 |
