# Supplementary material for: The Impact of Digital Health Interventions on Psychological Health, Self-Efficacy, and Quality of Life in Patients With End-Stage Kidney Disease: Systematic Review and Meta-Analysis
Source: J Med Internet Res. 2025 Sep 26;27:e74414. doi: 10.2196/74414 (PMC12466795; doi:10.2196/74414)
Supplement: Multimedia Appendix 2 [file jmir-v27-e74414-s002.doc]

| Outcomes | Risk of bias^a^ | Inconsistency^b^ | Indirectness^c^ | Imprecision^d^ | Other considerations^e^ | Certainty of the  evidence (GRADE)^f^ |
| --- | --- | --- | --- | --- | --- | --- |
| Psychological health |  |  |  |  |  |  |
| Depression | serious | serious | not serious | not serious | none | ⨁⨁◯◯ Low |
| Anxiety |  |  |  |  |  |  |
| General anxiety | serious | not serious | not serious | not serious | none | ⨁⨁⨁◯ Moderate |
| State anxiety | serious | not serious | not serious | very serious | none | ⨁◯◯◯ Very low |
| Trait anxiety | serious | not serious | not serious | very serious | none | ⨁◯◯◯ Very low |
| Stress | serious | not serious | not serious | very serious | none | ⨁◯◯◯ Very low |
| Self-efficacy | serious | serious | not serious | serious | none | ⨁◯◯◯ Very low |
| Quality of life |  |  |  |  |  |  |
| Quality of life（overall） | serious | serious | not serious | not serious | none | ⨁⨁◯◯ Low |
| Symptoms | not serious | not serious | not serious | very serious | none | ⨁⨁◯◯ Low |
| Physical Component Summary | serious | not serious | not serious | serious | none | ⨁⨁◯◯ Low |
| Mental Component Summary | serious | not serious | not serious | serious | none | ⨁⨁◯◯ Low |
| Pain | serious | not serious | not serious | serious | none | ⨁⨁◯◯ Low |
| Physical functioning | serious | not serious | not serious | very serious | none | ⨁◯◯◯ Very low |
| Physical role difficulty | serious | not serious | not serious | serious | none | ⨁⨁◯◯ Low |
| Emotional role difficulty | serious | not serious | not serious | very serious | none | ⨁◯◯◯ Very low |
| Vitality | serious | serious | not serious | very serious | none | ⨁◯◯◯ Very low |
| Mental health | serious | not serious | not serious | serious | none | ⨁⨁◯◯ Low |
| General health | serious | not serious | not serious | serious | none | ⨁⨁◯◯ Low |
| Social functioning | serious | serious | not serious | very serious | none | ⨁◯◯◯ Very low |

^a^ Risk of bias: Certainty was downgraded if more than 50% of the included studies were assessed as some concern/high-risk.

^b^ Inconsistency: Certainty was downgraded if *I*^2^ > 50%.

^c^ Indirectness: Refers to the extent to which the population, intervention, and outcomes of the included studies align with those of the target population and review. Studies involving significant differences in any of these factors were considered indirect and downgraded for this reason.

^d^ Imprecision: Assessed based on the optimal information size (OIS) and the width of the 95% confidence interval (CI). OIS for continuous outcomes was calculated as **4(*Z*_1-_*_α_*_/2_ + *Z*_1-_*_β_*)^2^*σ*^2^/*δ*^2^**, with *α* = 0.05, *β* = 0.20, *σ* = standard deviation from a relevant included study, and δ as the minimal clinically important difference(MCID). When studies used different measurement tools and standardized mean differences were pooled, OIS was estimated based on a selected instrument (one with an established MCID)[1]. If a study failed to meet the OIS, or if the CI was wide, we downgraded the evidence by one level. If the study did not meet the OIS and the CI was particularly wide, the evidence was downgraded by two levels.

^e^ Other considerations: Included publication bias, large effect, plausible confounding, and dose-response gradient. Evidence was evaluated and upgraded or downgraded accordingly to reflect their potential impact on the overall quality of evidence.

^f^ High certainty: further research is very unlikely to change our confidence in the estimate of effect; Moderate certainty: further research is likely to have an important impact on our confidence in the estimate of effect and may change the estimate; Low certainty: further research is very likely to have an important impact on our confidence in the estimate of effect and is likely to change the estimate; Very low certainty: any estimate of effect is very uncertain.
