# Supplementary material for: The Impact of Digital Health Interventions on Psychological Health, Self-Efficacy, and Quality of Life in Patients With End-Stage Kidney Disease: Systematic Review and Meta-Analysis
Source: J Med Internet Res. 2025 Sep 26;27:e74414. doi: 10.2196/74414 (PMC12466795; doi:10.2196/74414)

**1. Forest Plot Meta-Analyses for Different Outcomes**

**1.1. Forest Plot of DHIs Association with Depression**


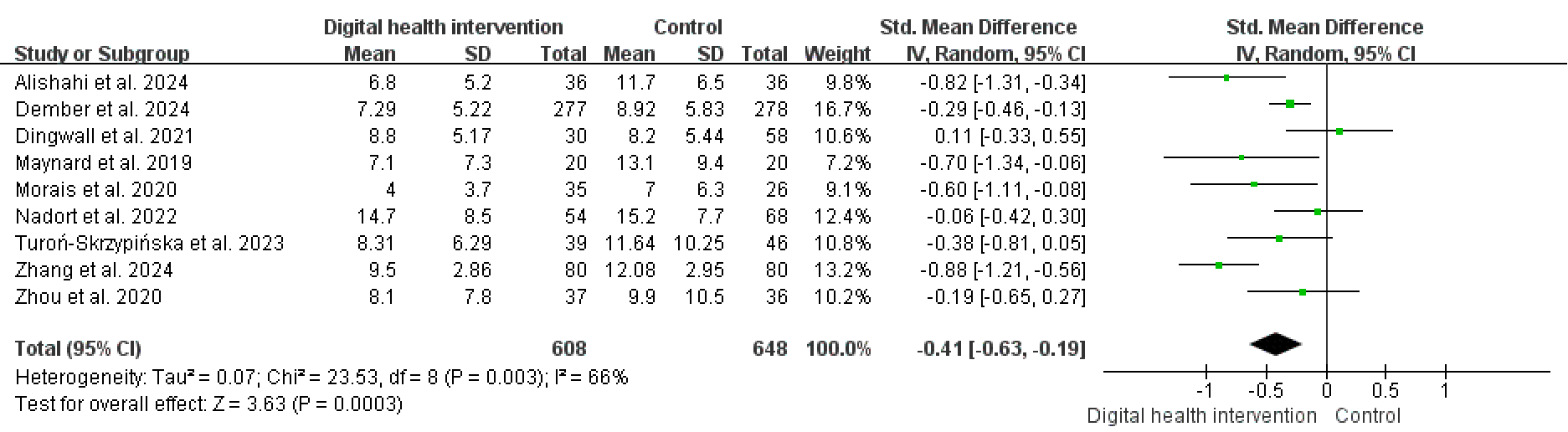


**1.2. Forest Plot of DHIs Association with General Anxiety**


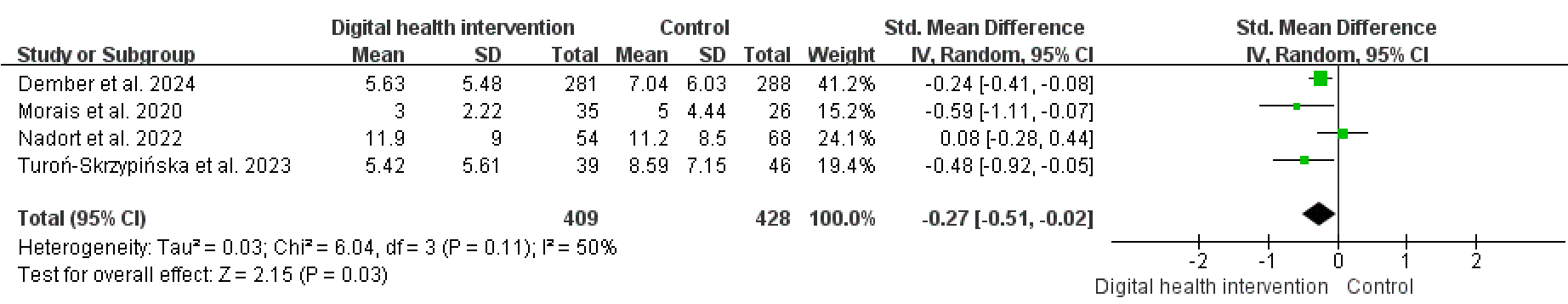


**1.3. Forest Plot of DHIs Association with State Anxiety**


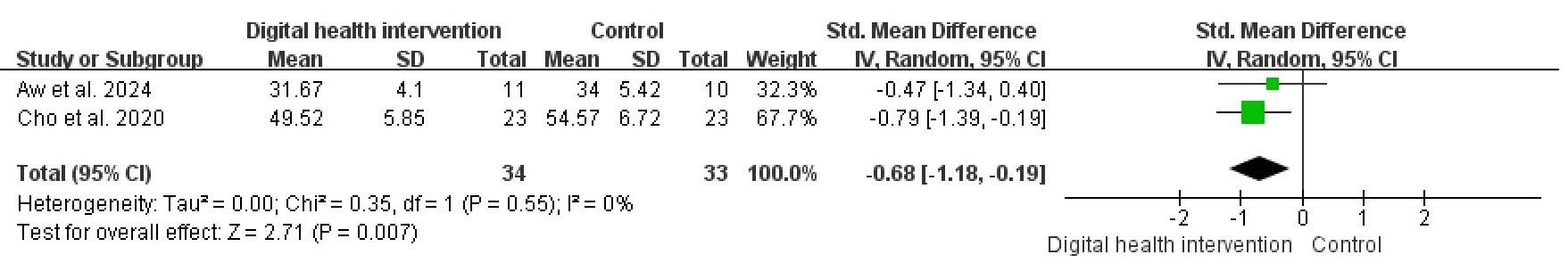


**1.4. Forest Plot of DHIs Association with Trait Anxiety**


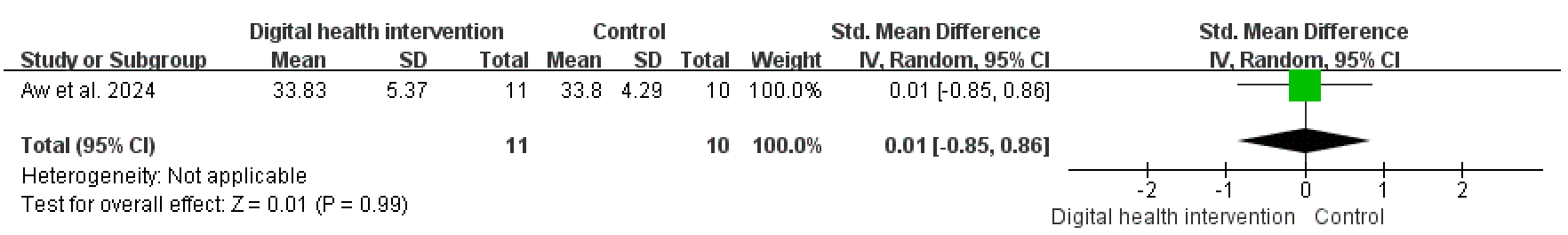


**1.5. Forest Plot of DHIs Association with Stress**


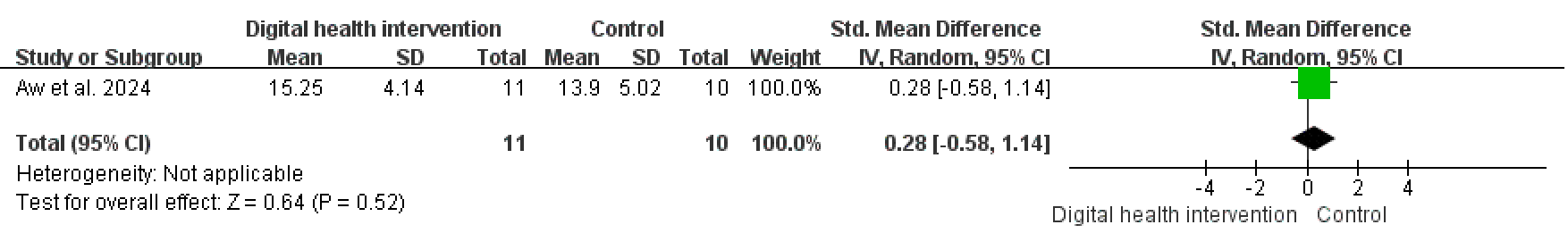


**1.6. Forest Plot of DHIs Association with Self-Efficacy**


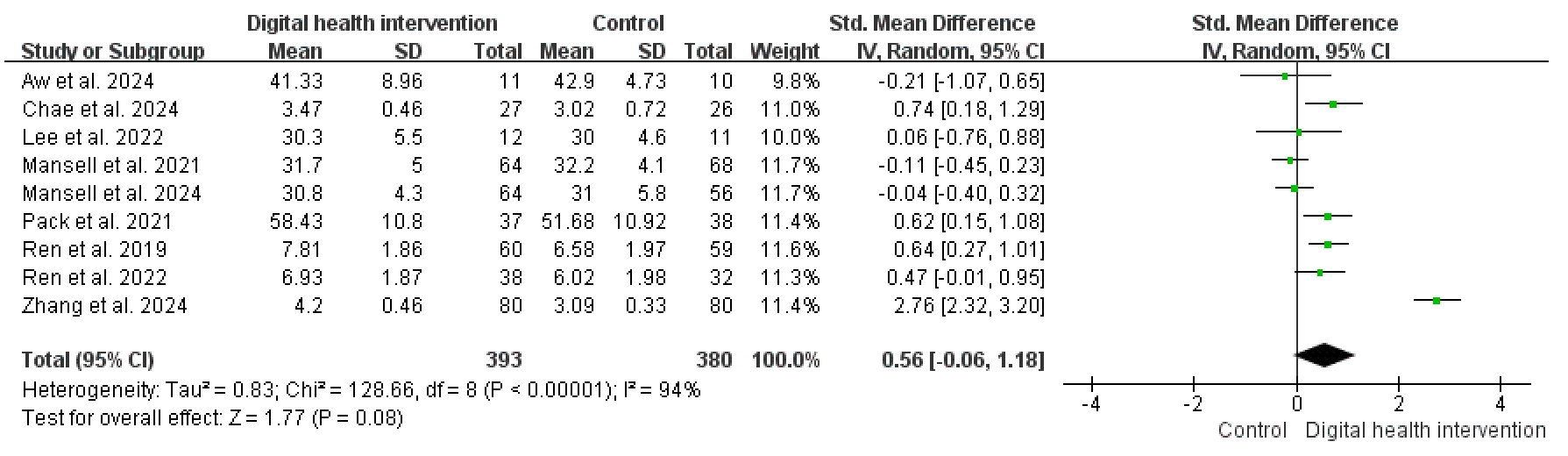


**1.7. Forest Plot of DHIs Association with Quality of life(Overall)**


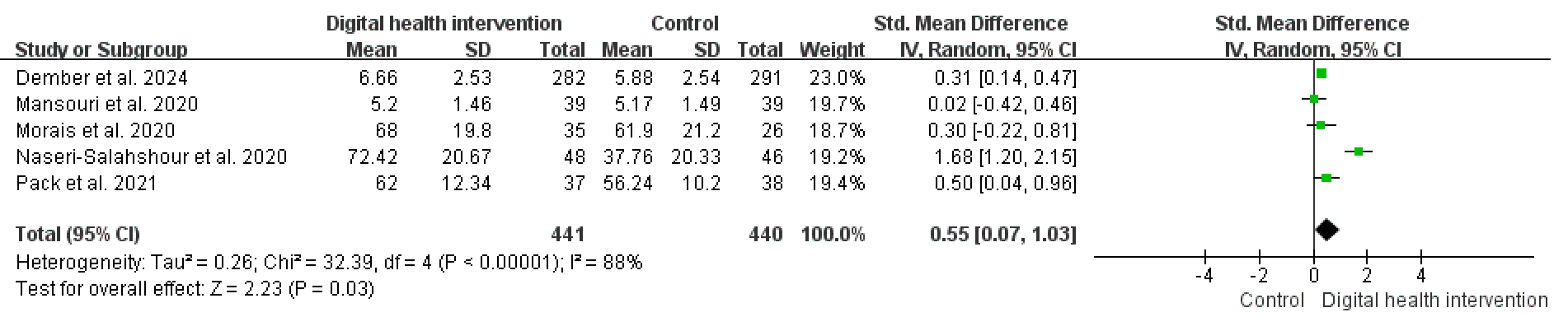


**1.8. Forest Plot of DHIs Association with Quality of life(Symptoms)**


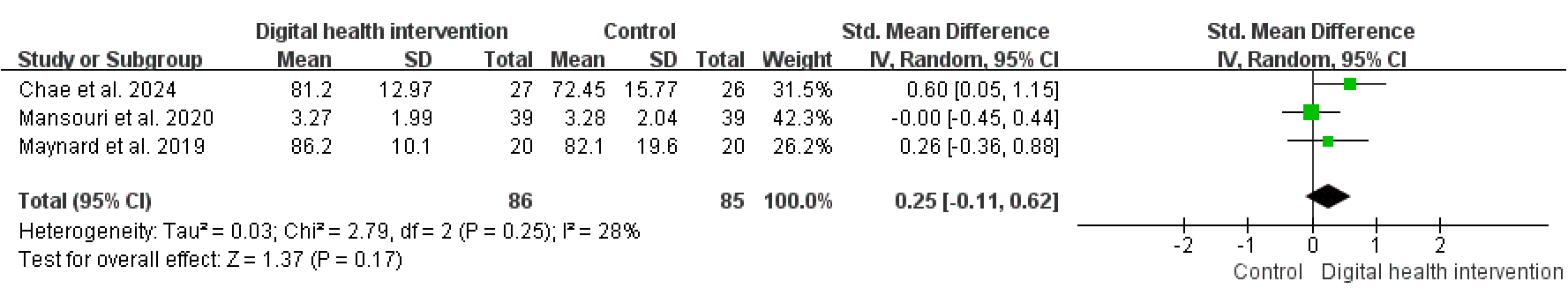


**1.9. Forest Plot of DHIs Association with Quality of life(Physical Component Summary)**


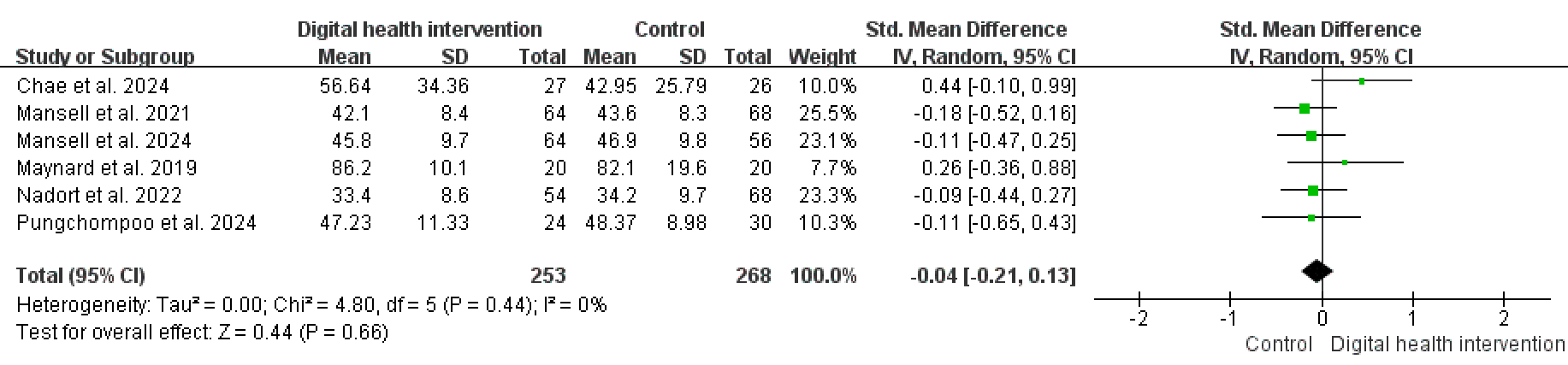


**1.10. Forest Plot of DHIs Association with Quality of life(Mental Component Summary)**


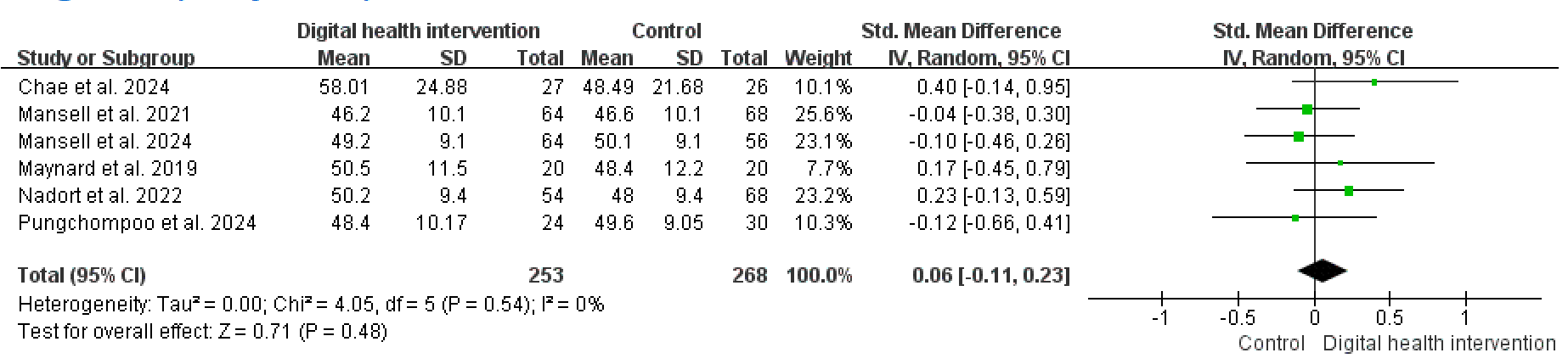


**1.11. Forest Plot of DHIs Association with Quality of life(Pain)**


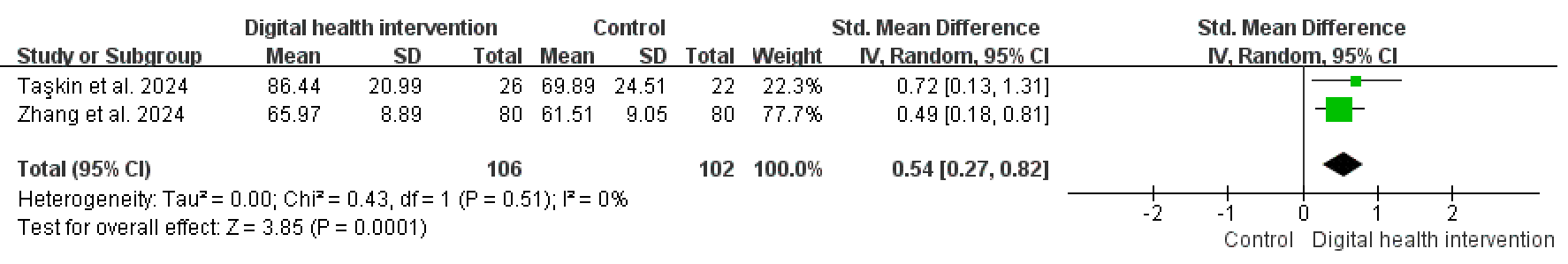


**1.12. Forest Plot of DHIs Association with Quality of life(Physical functioning)**


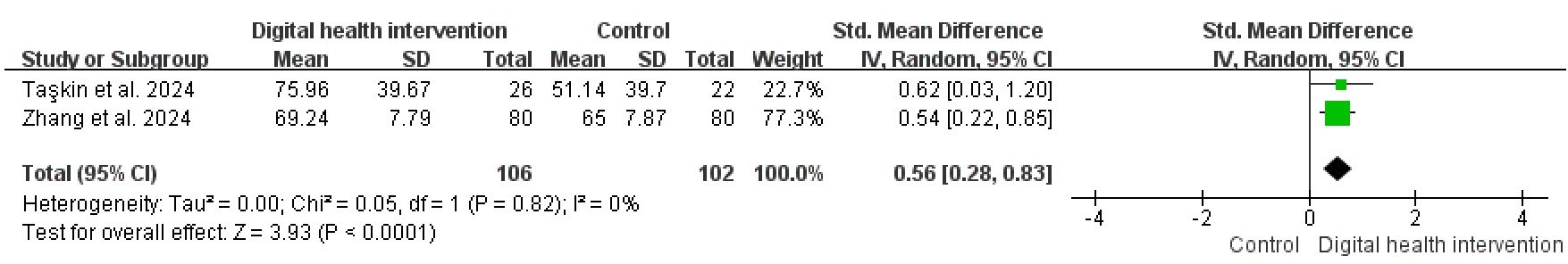


**1.13. Forest Plot of DHIs Association with Quality of life(Physical role difficulty)**


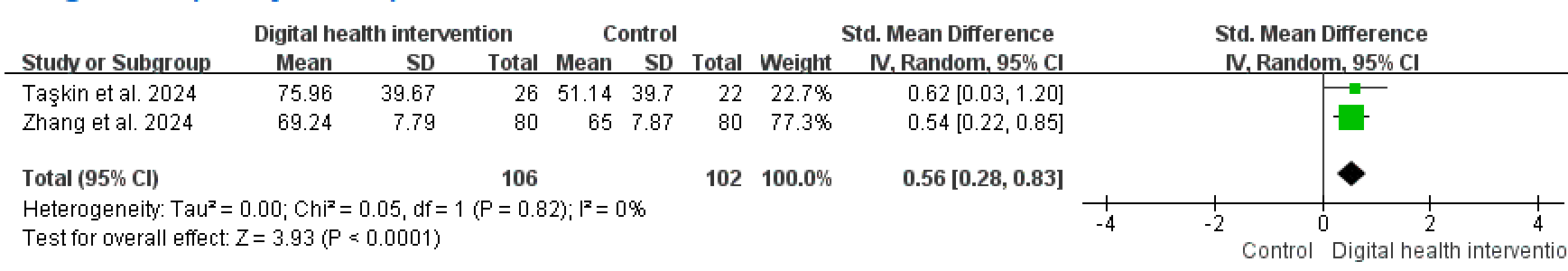


**1.14. Forest Plot of DHIs Association with Quality of life(Emotional role difficulty)**


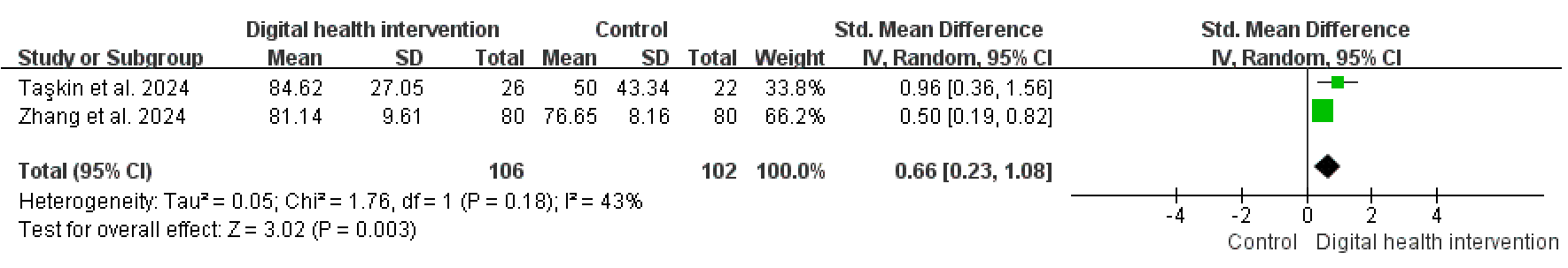


**1.15. Forest Plot of DHIs Association with Quality of life(Mental health)**


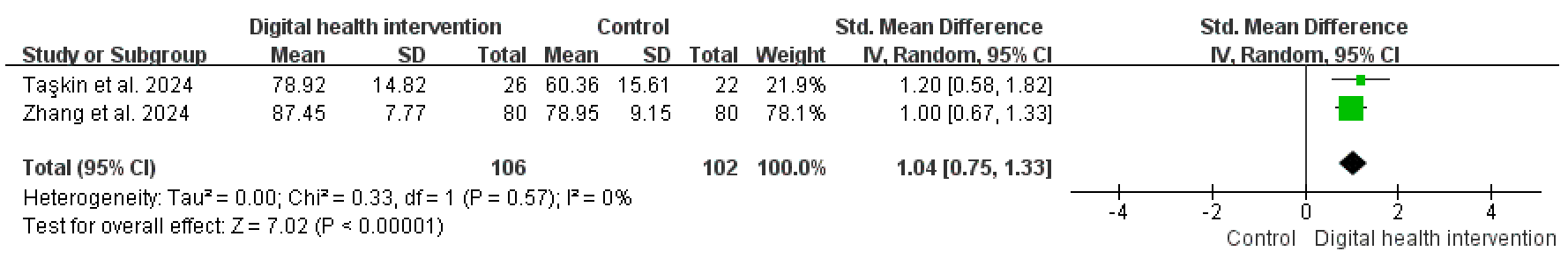


**1.16. Forest Plot of DHIs Association with Quality of life(General health)**


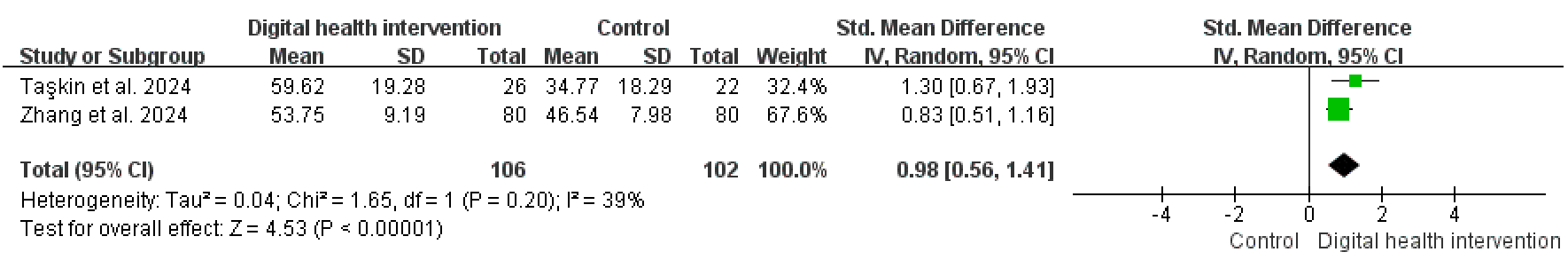


**1.17. Forest Plot of DHIs Association with Quality of life(Vitality)**


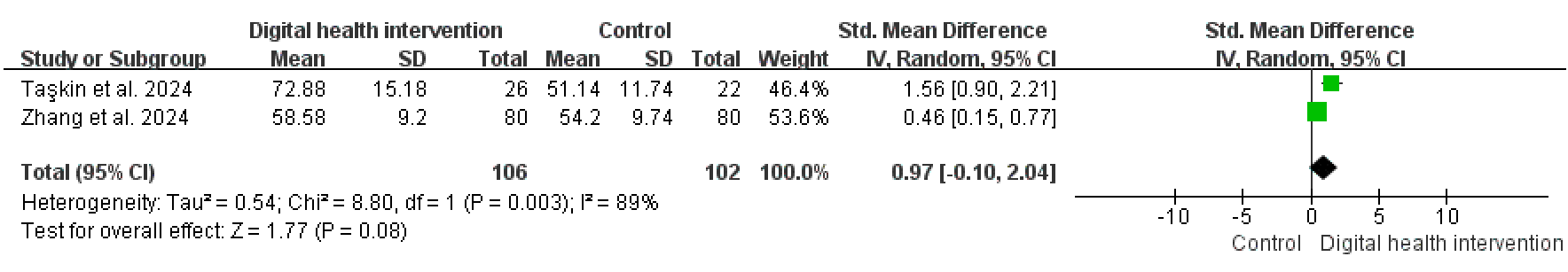


**1.18. Forest Plot of DHIs Association with Quality of life(Social functioning)**


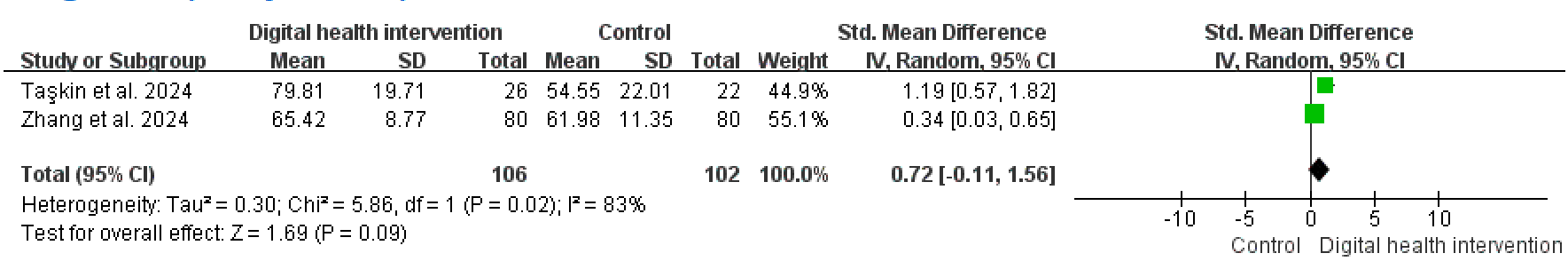


**2. Subgroup Analyses of Each Outcome According to Treatment**

**2.1. Subgroup Analysis of Depression According to Treatment**


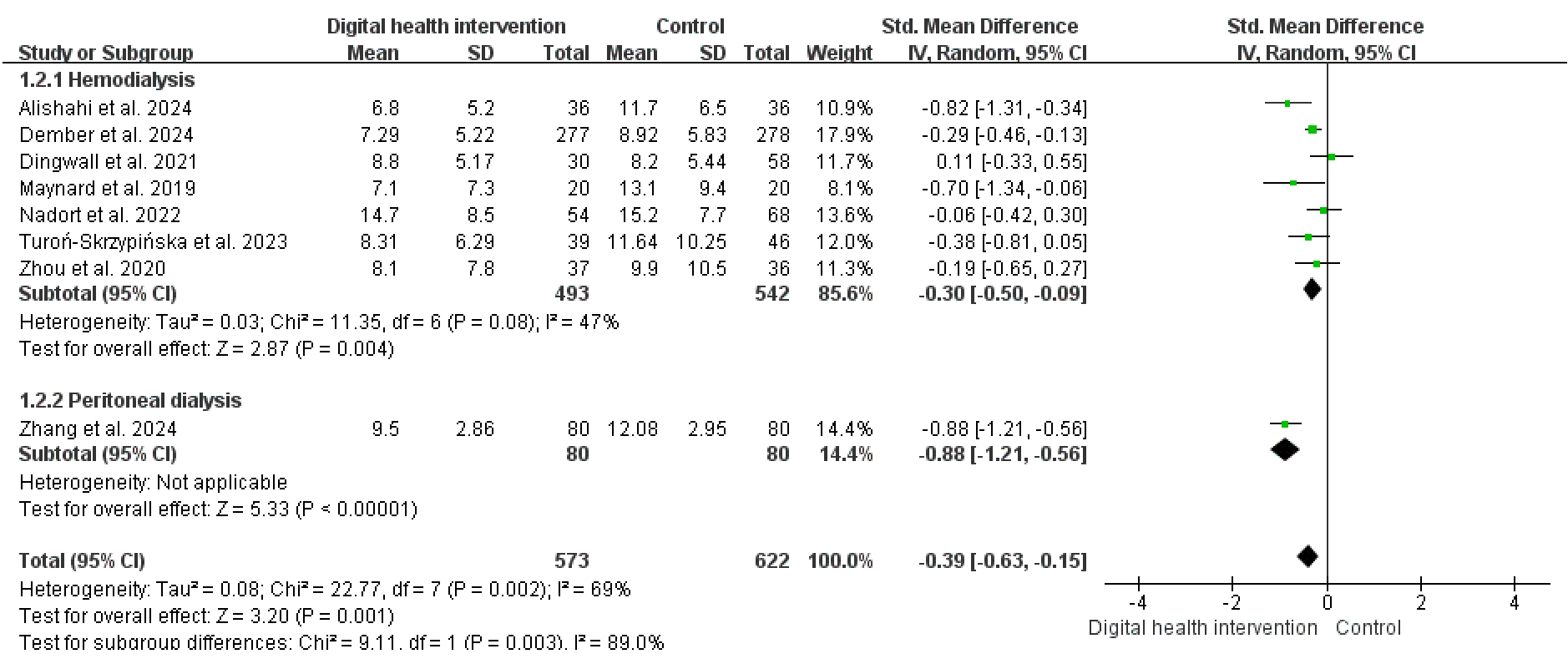


**2.2. Subgroup Analysis of Self-Efficacy According to Treatment**


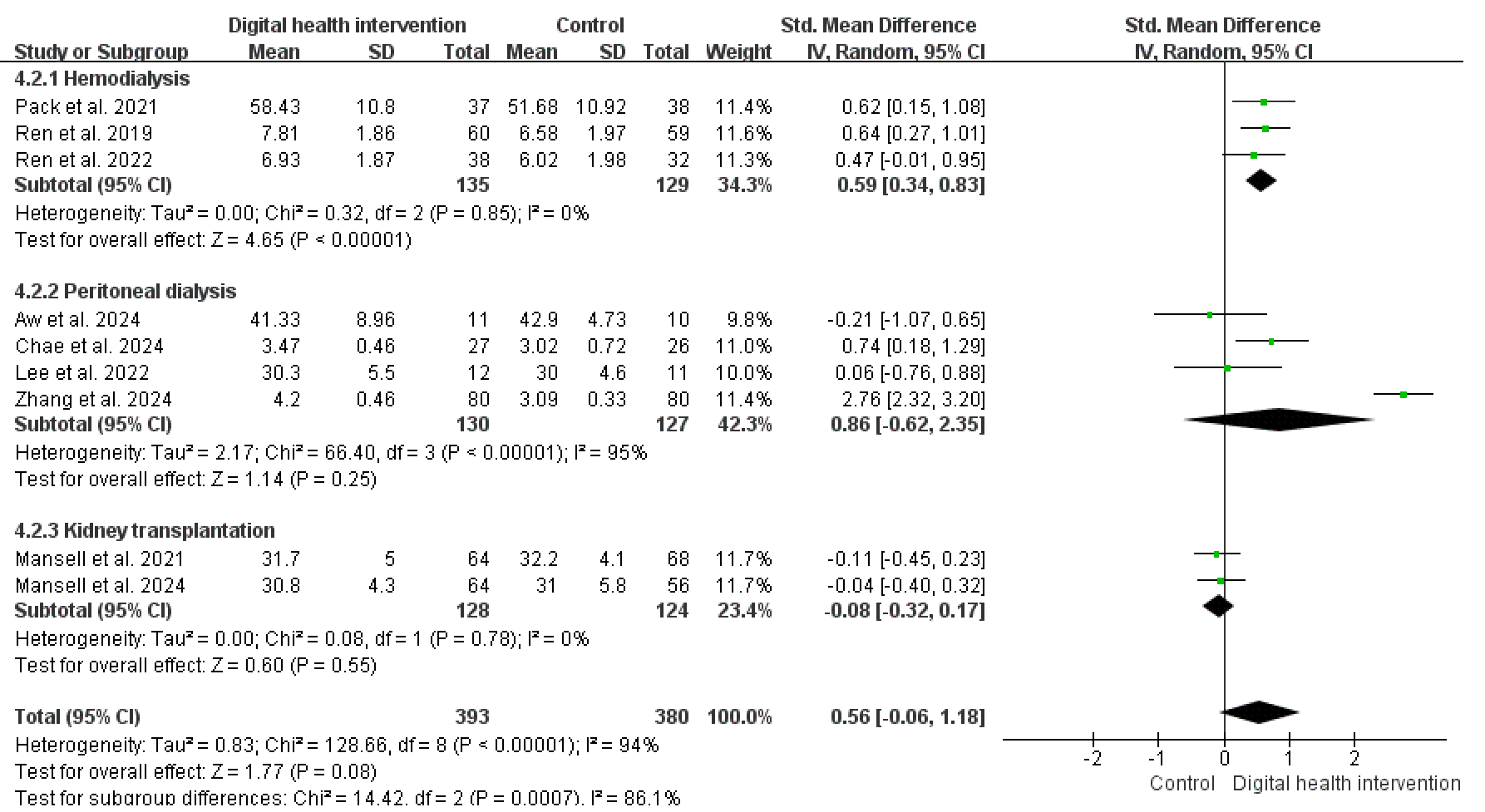


**2.3. Subgroup Analysis of Quality of Life According to Treatment**


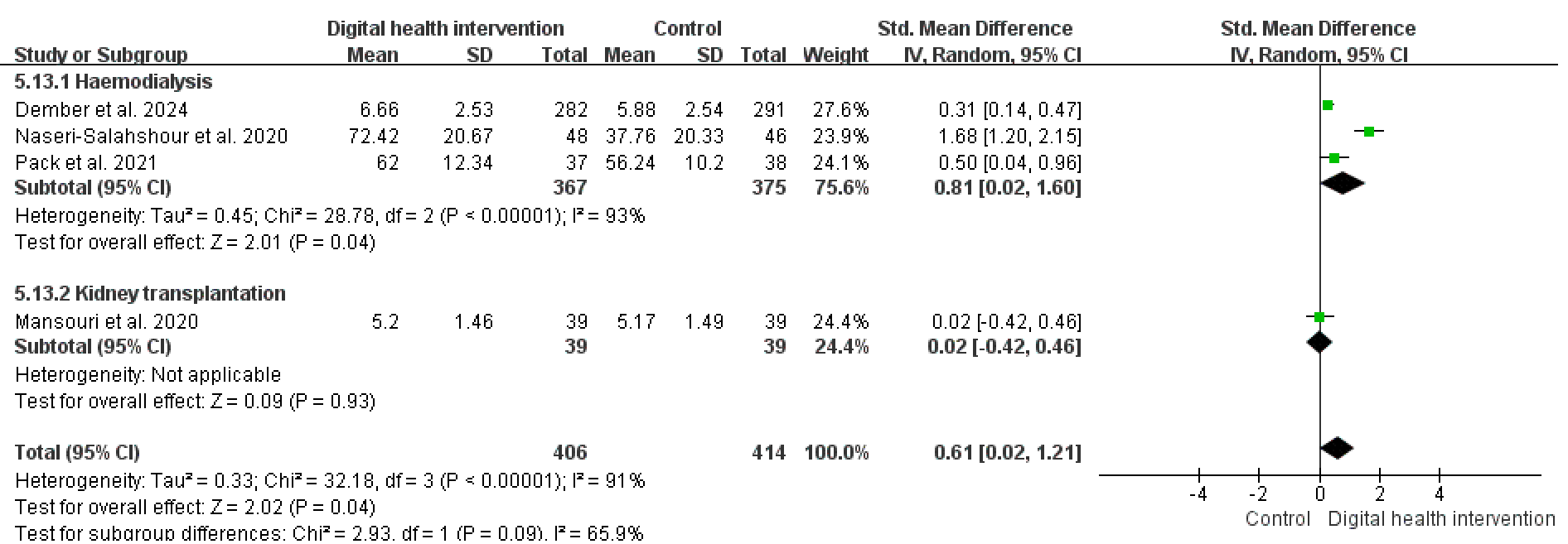


**3. Subgroup Analyses of Each Outcome According to Intervention Modality**

**3.1. Subgroup Analysis of Depression According to Intervention Modality**


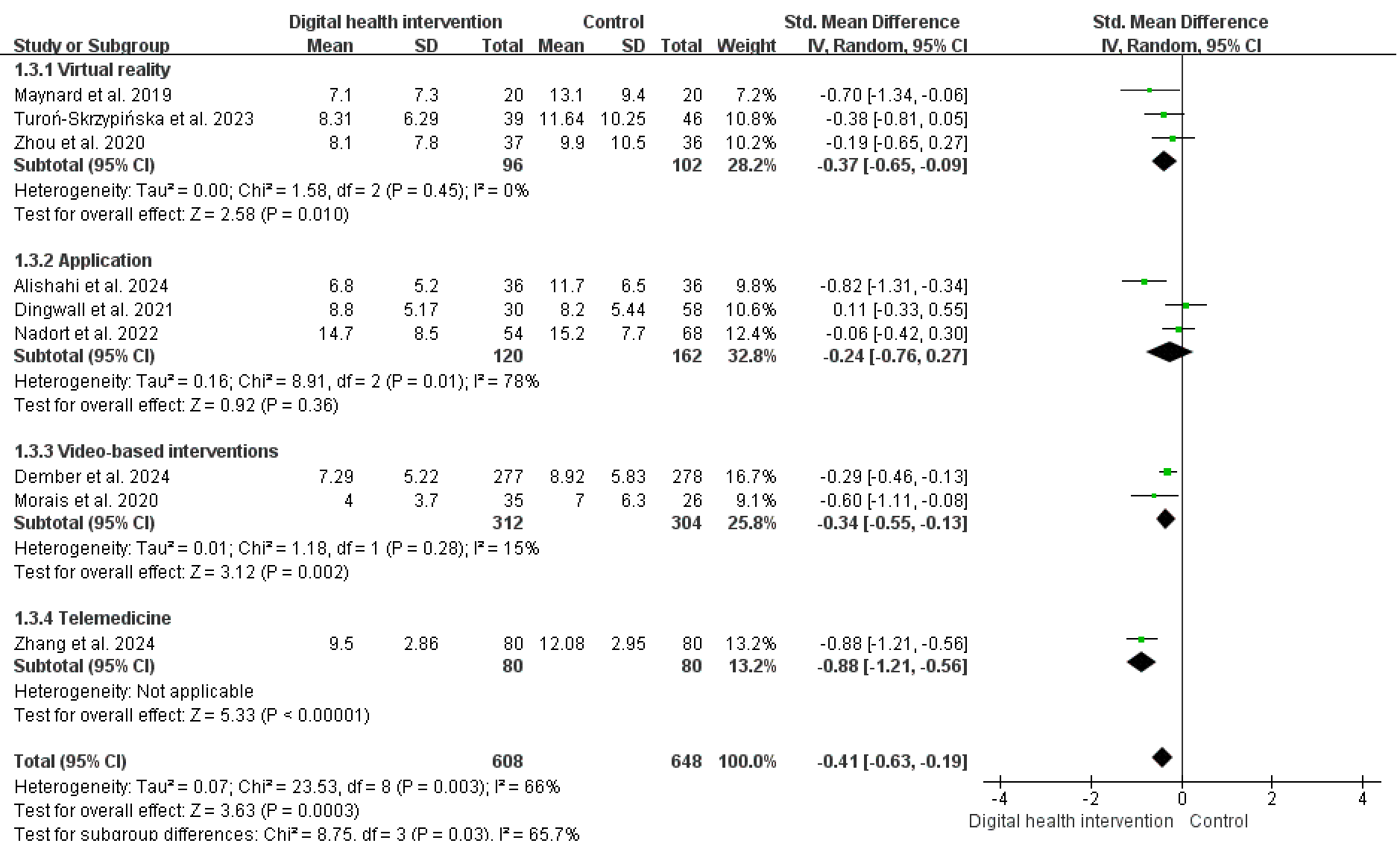


**3.2. Subgroup Analysis of Self-Efficacy According to Intervention Modality**


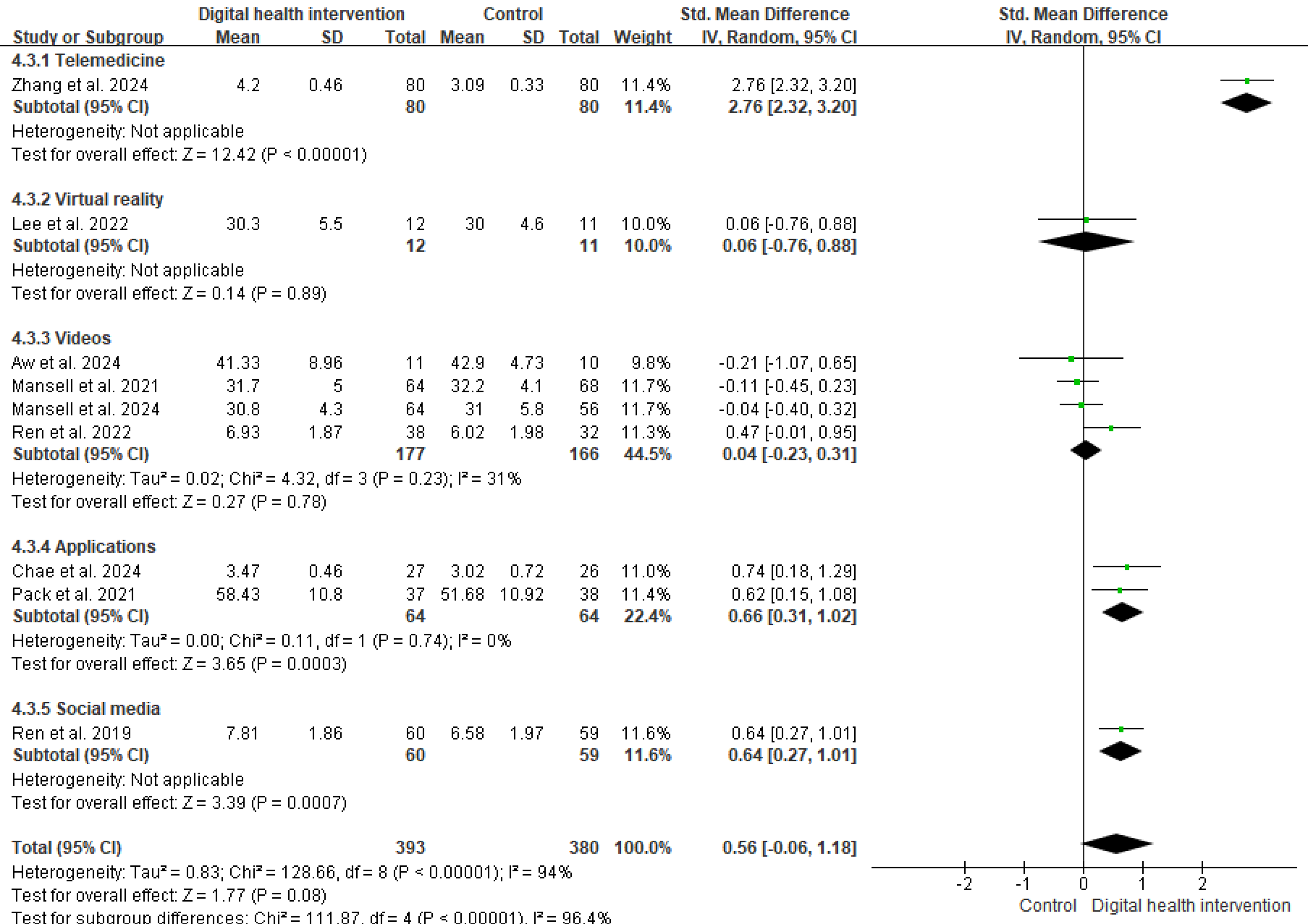


**3.3. Subgroup Analysis of Quality of Life According to Intervention Modality**


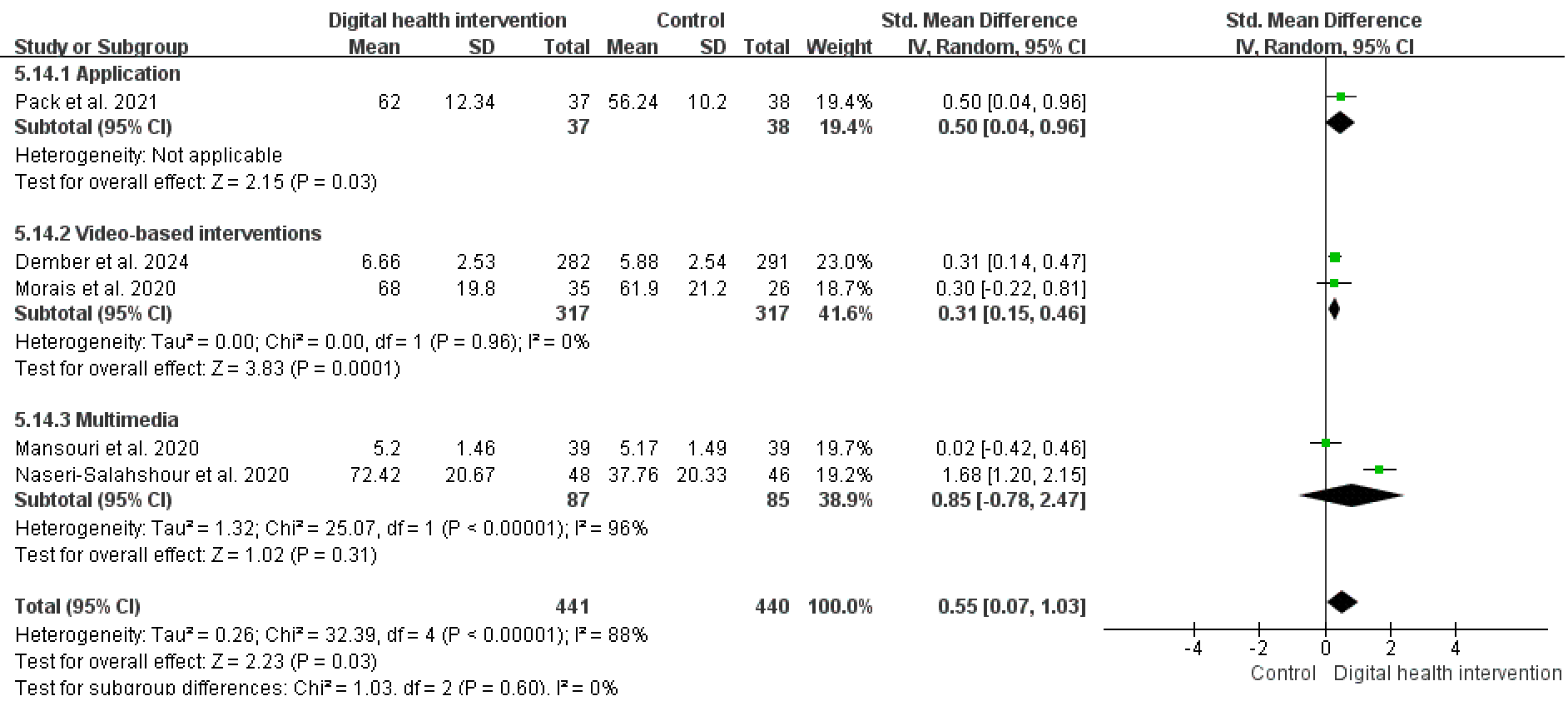


**4. Subgroup Analyses of Each Outcome According to Duration of Intervention**

**4.1. Subgroup Analysis of Depression According to Duration of Intervention**


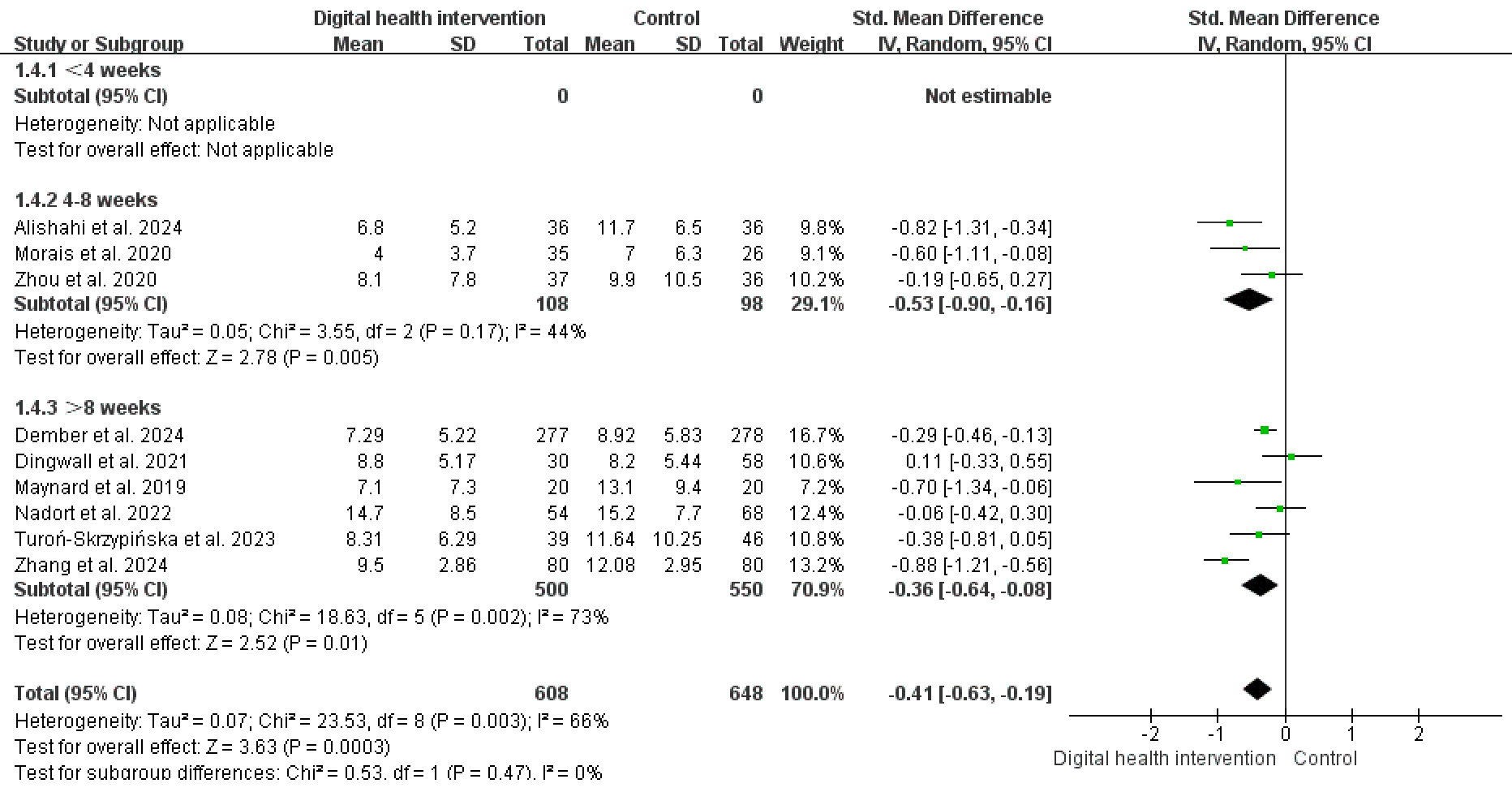


**4.2. Subgroup Analysis of Self-Efficacy According to Duration of Intervention**


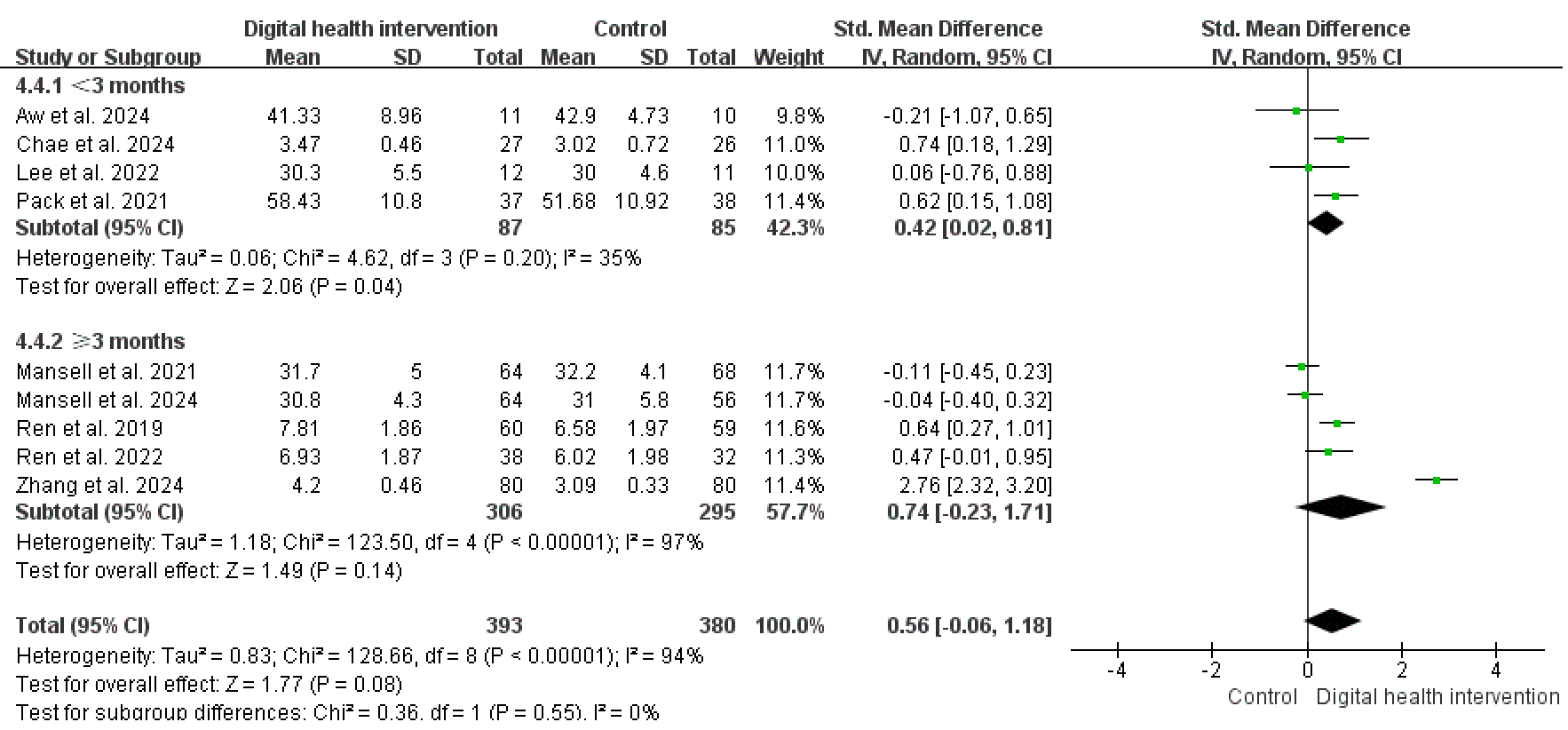


**4.3. Subgroup Analysis of Quality of Life According to Duration of Intervention**


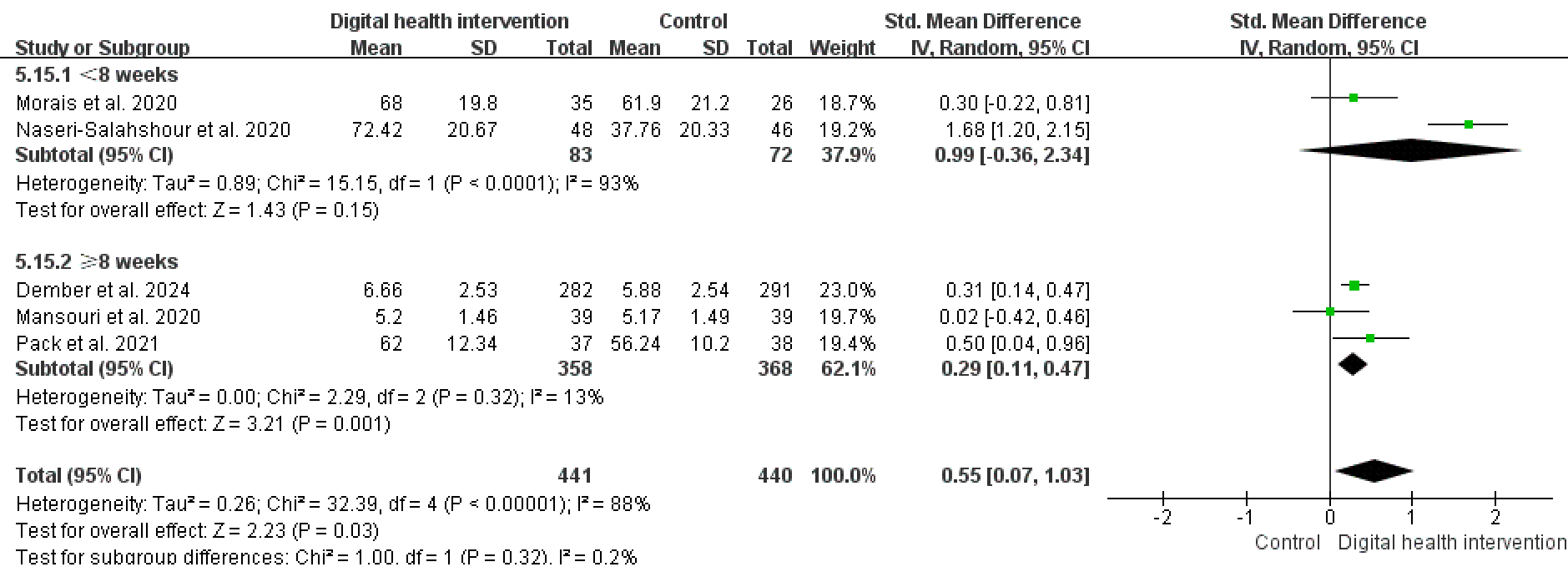


**5. Sensitivity Analysis**

**5.1 Sensitivity Analysis of General Anxiety Meta-Analysis: Forest Plot After Excluding the Study by Nadort**


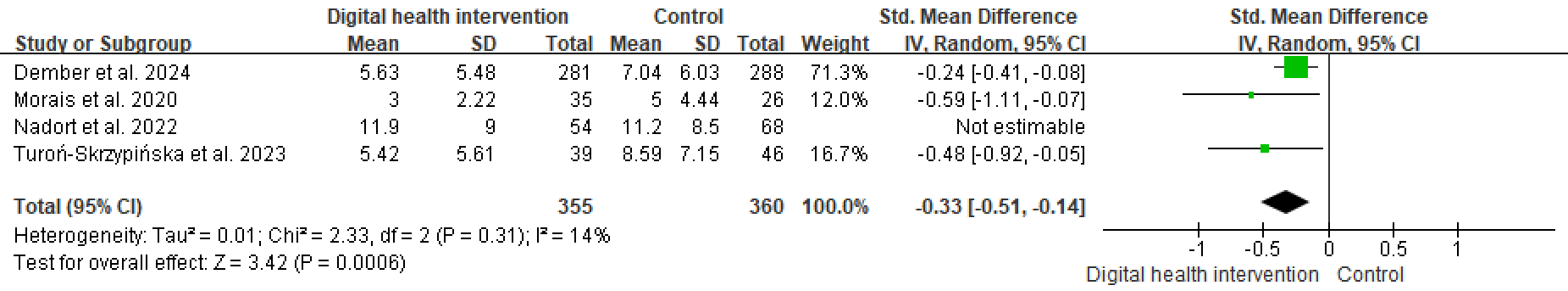


**5.2 Sensitivity Analysis of Self-Efficacy Meta-Analysis: Forest Plot After Excluding the Study with the Largest Weight**


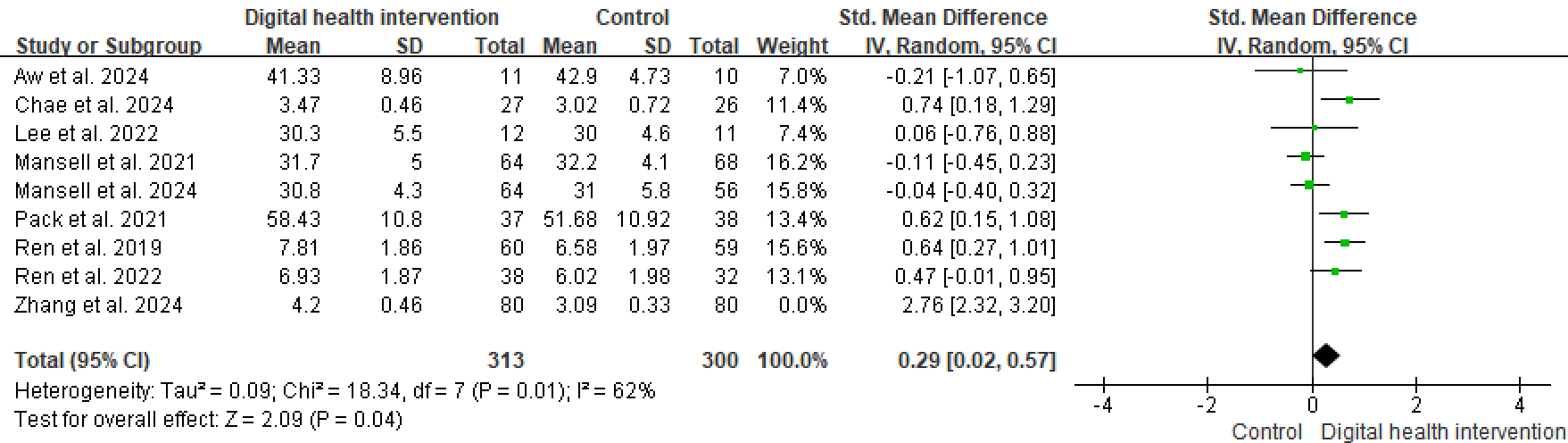


**5.3 Sensitivity Analysis of Overall Quality of Life Meta-Analysis: Forest Plot After Excluding the Study with the Largest Weight**


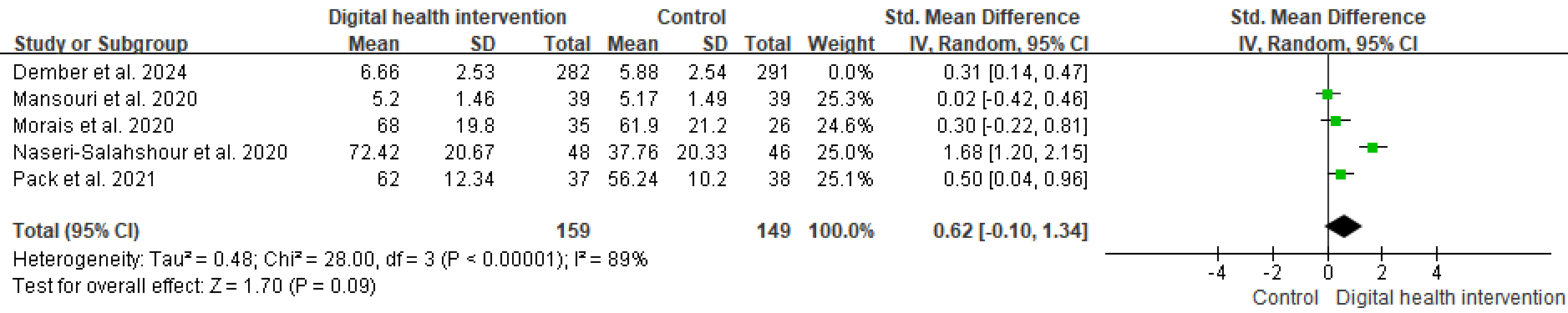

Supplement: Multimedia Appendix 3 [file jmir-v27-e74414-s003.doc]
